# Skill-Learning-Based Trajectory Planning for Robotic Vertebral Plate Cutting: Personalization Through Surgeon Technique Integration and Neural Network Prediction

**DOI:** 10.3390/biomimetics9120719

**Published:** 2024-11-21

**Authors:** Heqiang Tian, Xiang Zhang, Yurui Yin, Hongqiang Ma

**Affiliations:** College of Mechanical and Electronic Engineering, Shandong University of Science and Technology, Qingdao 266590, China; m15662399901@163.com (X.Z.); 18636838025@163.com (Y.Y.); hongqiangma2021@126.com (H.M.)

**Keywords:** robotic-assisted laminectomy, vertebral plate cutting, trajectory planning, skill learning, BP neural network

## Abstract

In robotic-assisted laminectomy decompression, stable and precise vertebral plate cutting remains challenging due to manual dependency and the absence of adaptive skill-learning mechanisms. This paper presents an advanced robotic vertebral plate-cutting system that leverages patient-specific anatomical variations and replicates the surgeon’s cutting technique through a trajectory parameter prediction model. A spatial mapping relationship between artificial and patient vertebrae is first established, enabling the robot to mimic surgeon-defined trajectories with high accuracy. The robotic system’s trajectory planning begins with acquiring point cloud data of the vertebral plate, which undergoes preprocessing, Non-Uniform Rational B-Splines (NURBS) fitting, and parametric discretization. Using the processed data, a spatial mapping method translates the surgeon’s cutting path to the robotic coordinate system, with simulation validating the trajectory’s adherence to surgical requirements. To further enhance the accuracy and stability of trajectory planning, a Backpropagation(BP) neural network is implemented, providing predictive modeling for trajectory parameters. The analysis and training of the neural network confirm its effectiveness in capturing complex cutting trajectories. Finally, experimental validation, involving an artificial vertebral body model and cutting trials on patient vertebrae, demonstrates the proposed method’s capability to deliver enhanced cutting precision and stability. This skill-learning-based, personalized trajectory planning approach offers significant potential for improving the safety and quality of orthopedic robotic surgeries.

## 1. Introduction

In recent years, the incidence of spine-related conditions has steadily risen, largely due to spinal nerve compression. Laminectomy and decompression surgery are among the most effective treatments for this condition, with the quality of the laminectomy playing a crucial role in determining the surgical outcomes. This procedure requires the surgeon to manipulate surgical instruments to cut through one or both sides of the spinal spinous process. The cutting trajectory is heavily influenced by the surgeon’s experience, and prolonged cutting can lead to wrist fatigue, which may result in hand tremors and increase the risk of injury to surrounding blood vessels or nerves [1].

The integration of robotic technology has significantly enhanced traditional spinal surgery techniques, aiding surgeons in performing precise vertebral plate-cutting procedures [2,3]. However, as clinical trials advance and individualized variations in patients’ vertebral anatomy become more evident, there is an increasing need for robots to learn surgical skills from the motion trajectories demonstrated by experienced surgeons. This learning is essential, as it enables robots to adapt to personalized surgical requirements, ensuring stability and safety throughout the nonlinear and unstructured vertebral plate-cutting process. Conventional orthopedic robots primarily rely on Computed Tomography (CT) imaging navigation systems for preoperative planning to generate cutting trajectories. However, the development of personalized cutting trajectories remains a challenge due to the lack of a surgical skill-learning mechanism and preoperative simulation that are tailored to the specific needs of individual patients. This limitation underscores the necessity for innovative approaches in robotic skill learning, as well as the integration of personalized surgical data into preoperative planning, which is crucial for enhancing surgical precision. The absence of such systems compromises the accuracy of preoperative diagnoses in high-risk orthopedic surgeries and diminishes the cutting accuracy and safety during intraoperative robotic procedures. Consequently, the development of precise positioning and cutting techniques must incorporate advanced preoperative planning and skill-learning methods to ensure optimal surgical outcomes.

Recent research has increasingly focused on methods for planning personalized vertebral plate-cutting trajectories, highlighting the importance of integrating skill-learning techniques into robotic systems. However, the application of these methods remains limited, particularly in clinical settings where individualized approaches are essential for optimizing surgical outcomes. Developing skill-learning-based strategies for personalized cutting trajectory planning has the potential to minimize damage to skeletal tissues while improving cutting accuracy and efficiency, thereby adding significant value to clinical research. Globally, researchers have conducted extensive studies on trajectory planning for orthopedic robots. CT imaging, which contains rich medical data, allows for the precise localization of lesions and facilitates the planning of surgical paths after systematic analysis and processing. In contrast to traditional manual methods, CT images mitigate the influence of subjective experience and external environmental factors, positioning them as a critical tool in assisting surgeons with planning cutting trajectories for orthopedic robots [4,5]. Guven et al. [6] performed bone-cutting operations by defining the cutting trajectory within the CT image space, using a medical user interface to translate these images into the robot’s operational space. Woo et al. [7] employed CT medical images for virtual surgical planning and motion analysis around anatomical landmarks, enabling automatic repositioning during the planning phase. Ding and Sun [8,9] proposed a robotic cutting path planning method based on 3D medical images, correlating the surgeon’s planned cutting trajectory with the robot through human–robot interaction and 3D reconstruction, thus facilitating robot-assisted spine plate cutting and decompression surgery. Malinda et al. [10] developed a fully automated spine segmentation method using CT images by combining Convolutional Neural Networks (CNNs) and Fully Convolutional Networks (FCNs), leveraging class redundancy as a soft constraint to improve segmentation accuracy. This advancement is crucial for diagnosing spinal disorders and performing surgeries with computer-assisted systems. Sugita et al. [11] introduced a geometric optimization method to minimize the risk of collision between the cutting tool and soft tissue, constructing a cutting model that represents the tool, interference zone, opening zone, and resection zone, leading to high-precision, collision-free tool paths. Abraham et al. [12] developed a Cartesian space-based robot cutting trajectory algorithm that automatically computes a path covering the bone surface. This algorithm integrates cutting trajectories with scanning lines and potential functions to generate uniformly distributed cutting paths. Federspil et al. [13] investigated bone cutting for ear neurosurgery and proposed a spiral horizontal cutting method, implementing it using a six-degree-of-freedom robotic platform, thereby refining the robotic bone layer cutting trajectory planning technique.

With advancements in robotics and artificial intelligence technology, robot skill learning has emerged as a key research area. This includes the development of techniques allowing robots to learn from expert demonstrations and adapt to diverse anatomical conditions, a step crucial for their clinical application in surgery. In response to the practical application needs of robots across diverse task scenarios, researchers worldwide have developed a variety of robot skill-learning methods leveraging deep learning, reinforcement learning, and demonstration learning. These approaches have yielded significant advancements, particularly in robot-assisted surgery and related fields. Tian et al. [14] proposed an efficient motion-grinding strategy for spinal surgery robots, where the robotic vertebral plate-grinding trajectory is planned by recording the surgeon’s cutting path. This method minimizes trauma to the patient’s bone tissue while improving both the precision and efficiency of the bone-grinding process. Dong et al. [15] introduced a deep learning-based imitation learning framework to enhance robotic motion imitation by establishing biomechanical relationships between models. Li et al. [16] applied deep neural networks, trajectory generation strategies, and sanding speed adjustment techniques to vertebral plate localization. By extracting surgical information from CT images, these algorithms automate sanding trajectory planning, thereby simplifying the surgical workflow and reducing the manual workload for surgeons. Huang et al. [17] proposed an interactive approach to collect expert demonstration data by physically dragging the robotic arm. This method enables experts to perform a range of tasks in demonstration mode, capturing data such as the robot’s trajectory and speed. Koenemann et al. [18] utilized wearable sensor devices to gather human movement data, allowing a humanoid robot to replicate complex full-body human motions in real-time. Similarly, Zhang et al. [19] developed a joystick-based teleoperation system that enables the remote control of a robot to perform specific tasks and collect real-time demonstration data. Another approach to expert data collection involves visual interaction; for example, Wang et al. [20] proposed a method for gathering expert hand movement information through visual feedback, enabling remotely operated manipulators to grasp objects with varying physical properties. Li et al. [21] employed a somatosensory camera to capture and model expert movements, generalizing these actions and completing the learning process with a trajectory generation algorithm. Reinhold et al. [22] introduced an image-based automation technique for uniform motion along arbitrarily shaped surfaces. This technique, designed by a physician and executed by a robot, improves the accuracy of surface medical procedures, such as precise cutting or tissue removal in spinal stenosis surgeries. Finally, Qin et al. [23] proposed a collaborative two-robot system for orthopedic surgery navigation. This system features an additional robot that actively adjusts the optical tracking system’s observation angle and incorporates collaborative preoperative planning, covering both the surgical robot’s osteotomy path and the navigation robot’s plan.

This paper addresses the current clinical demands of surgery and the emerging trends in robot-assisted surgical technology, with a particular focus on the challenge of vertebral plate cutting in orthopedic robotic surgery. Incorporating recent advancements in skill learning and trajectory planning, this study proposes a novel approach that integrates surgeon-specific cutting techniques into the robotic system, enhancing its ability to perform personalized and precise surgeries. To improve the robot’s ability to execute personalized vertebral plate cutting, an optical positioning system is employed to establish a spatial mapping relationship for the cutting trajectory, thereby emulating the surgeon’s manual cutting technique. The process involves fitting the cutting trajectory point cloud, among other steps, in the planning of robotic vertebral plate cutting. By mapping the relationship between manual and robotic cutting trajectories, the surgeon’s personalized cutting path is transferred into the robotic system, thereby integrating the surgeon’s expertise and operational skills into the robotic platform. Building on this approach, a BP neural network model is developed and combined with trajectory feature parameters to predict the vertebral plate-cutting trajectory. Finally, using the registration mapping relationship established between the artificial and patient-specific vertebral bodies during the operation, traditional virtual space image navigation technology is transformed into real-space navigation technology. This adaptation, based on the surgeon’s cutting trajectory and skill learning, enables the robot to inherit the surgeon’s operational skills through surgical simulation. As a result, the robot transitions from a simple operation-based system to a skill-based system, fully replicating the surgeon’s cutting techniques.

The contributions and innovations of this paper are centered around the development of a spatial mapping relationship that allows the robot to accurately replicate the surgeon’s manual cutting trajectory by translating it into the robot’s coordinate system. Unlike traditional methods, the proposed trajectory planning approach integrates a BP neural network to predict personalized cutting trajectories. This model enhances surgical adaptability while minimizing the need for large-scale datasets and real-time feedback. By generating smooth trajectory points, the BP neural network improves cutting accuracy and stability, while precisely predicting cutting parameters to account for individual anatomical variations. Furthermore, by aligning artificial and patient vertebrae through anatomical landmarks and registration matrices, the method effectively reduces trajectory transformation errors. The experimental validation of the proposed robotic trajectory planning method demonstrates its superior performance in enhancing cutting precision and stability, both in artificial vertebral models and patient vertebrae. The results indicate that the method outperforms traditional robotic orthopedic surgery approaches in terms of accuracy, adaptability, and efficiency.

The structure of this paper is as follows: Section 2 outlines the Materials and Methods, describing the establishment of spatial mapping relationships for the robotic vertebral plate-cutting system, including the acquisition and preprocessing of point cloud data, cutting point cloud fitting with NURBS curves, and the generation of robotic cutting trajectories. This section also discusses the BP neural network-based trajectory parameter prediction and the registration process for aligning artificial vertebrae with patient-specific vertebrae. Section 3 presents the Results and Discussion, which includes the accuracy validation of the spatial mapping relationship, simulation verification of trajectory planning based on the surgeon’s trajectory, trajectory planning simulations with parameter predictions, and experimental results of the robotic vertebral plate-cutting trajectory planning. This study lays a solid foundation for personalized skill learning in orthopedic robotic systems, offering a novel approach with potential clinical applications and significant improvements in surgical outcomes.

## 2. Materials and Methods

### 2.1. Establishment of Spatial Mapping Relationship for Robotic Vertebral Plate-Cutting System

**Figure 1:**
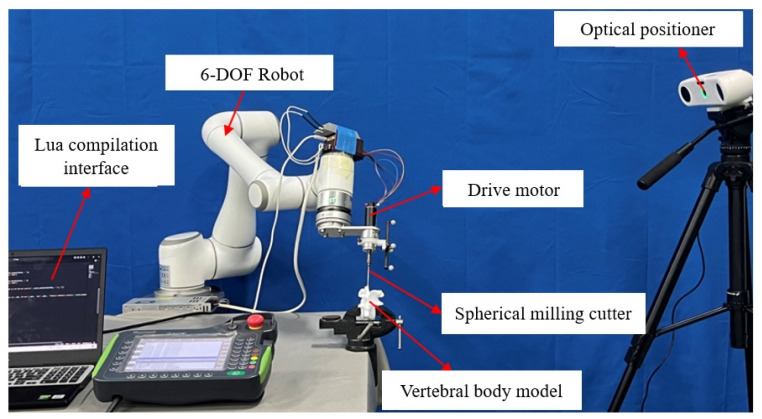
Robotic platform for vertebral plate cutting.

The robotic vertebral plate-cutting platform presented in this study is designed to replicate the surgeon’s personalized cutting trajectory for vertebral plate surgery. As illustrated in Figure 1, the system comprises several key components: a six-degree-of-freedom robot, an optical positioning system, a bone-grinding drill, and an artificial vertebral body model. The robot is equipped with six degrees of freedom, allowing for versatile and precise movement. It has a payload capacity of 6 kg, a maximum speed of 2800 mm/s, a maximum workspace of 914 mm, and a repeat positioning accuracy of ±0.03 mm. The AP-STD-100 optical positioning system is integrated into the platform, offering a maximum update frequency of 60 Hz and positioning accuracy of 0.12 mm. This system uses a passive positioning tool that employs an optical marker sphere for system identification, enabling the real-time tracking of tool position and orientation data. The bone-grinding drill consists of a single-flute medical ball milling cutter and a drive motor. The cutting tool has a ball diameter of 4 mm, a shaft diameter of 2.35 mm, and an overall length of 70 mm, fabricated from cemented carbide to ensure durability and precision. The drive motor, a DC brushless AM-BL2453AE model, has a maximum no-load speed of 28,715 rpm, a maximum rated torque of 12.4 Nm·m, and a maximum output power of 257.5 W. The artificial vertebral body model used in this platform is constructed from patient-specific CT scan data, which is processed through modeling and 3D printing. This model closely mimics the surface characteristics of real bone vertebrae, making it suitable for subsequent validation experiments.

**Figure 2:**
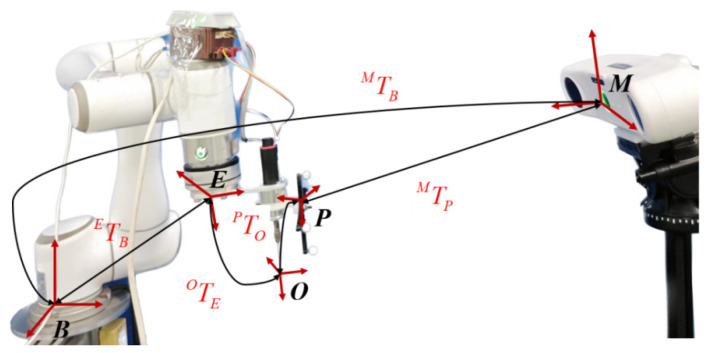
Spatial mapping relationship of optical measurement systems.

In the process of vertebral plate cutting and machining, the robot must acquire surgical operation skills from the motion trajectory demonstrated by the surgeon during preoperative cutting simulations in order to generate a personalized cutting trajectory for the patient. The primary objective is to establish a mapping relationship between the optical measurement space and to complete the registration between the robot and surgeon trajectories. This optical measurement space mapping relationship, as illustrated in Figure 2, involves several coordinate systems: the robot base coordinate system $\left\{B\right\}$, the robot flange coordinate system $\left\{E\right\}$, the optical tracking marker coordinate system $\left\{P\right\}$, the optical measurement coordinate system $\left\{M\right\}$, and the surgical instrument coordinate system $\left\{M\right\}$. Each of these coordinate systems plays a critical role in accurately mapping the surgeon’s cutting trajectory to the robot, ensuring that the robot can faithfully replicate the surgeon’s skills and achieve precise cutting during the surgical procedure.

In Figure 2, the relationship TBE can be established through robot kinematic analysis, while TPM can be obtained through real-time tracking using an optical positioner. To achieve a closed-loop transformation among all the coordinate systems shown in the diagram, it is critical to determine the transformation matrix for the remaining unknown coordinate systems. This step is essential for ensuring accurate spatial alignment and seamless integration between the various components of the robotic system.

(1) Solution of the transformation matrix TEO

In this paper, a tool calibration method is employed to calibrate the coordinate system of the robotic surgical instruments and determine the corresponding transformation matrix TEO. The calibration process consists of two main steps: position calibration and pose calibration.

**Figure 3:**
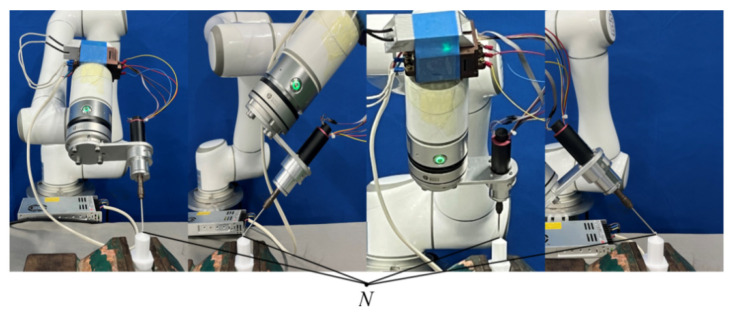
Calibration process for position and pose.

For position calibration, the “four-point method” is utilized. This method requires the spherical milling cutter to contact the reference point *N* on the calibration block at four distinct positions. As illustrated in Figure 3, this process ensures that the position of reference point *N* remains constant throughout the calibration, allowing for the precise determination of the surgical instrument’s position relative to the robot’s coordinate system. By accurately calibrating both the position and pose of the surgical instruments, a reliable transformation matrix is established, integrating the various coordinate systems into a unified framework. This enables the robot to precisely replicate the surgeon’s cutting trajectory, ensuring accurate and efficient performance during surgery.

The transformation relationship between the surgical instruments at the four reference points and the robot’s base coordinate system is represented by Equation (1) during the position calibration, which involves four position and pose transformations. These transformations are essential for accurately aligning the surgical instruments’ coordinate system with the robot’s base coordinate system, ensuring precise tool positioning during the surgical procedure.

(1) (TBO)i=(TBE)i·TEO

The preceding equation leads to Equation (2), which defines the relationship between the coordinate systems during the calibration process:

(2) (RBO)i(PBO)i01=(RBE)iREO(RBE)iPEO+(PBE)i01

In Equation (2), R denotes the rotation matrix between the coordinate systems and P denotes the translation matrix between the coordinate systems. Decomposing the last column of Equation (2) gives us the following equation:

(3) (PBO)i=(RBE)iPEO+(PBE)i

Since the position of the reference point *N* of the calibration block remains fixed, PBO is a definite constant value. Combining the above equations leads to Equation (4):

(4) (RBE)1−(RBE)2(RBE)2−(RBE)3(RBE)3−(RBE)4×PEO=(PBE)2−(PBE)1(PBE)3−(PBE)2(PBE)4−(PBE)3

This equation represents a system of linear equations with an irreducible coefficient matrix. To solve for the transformation parameters, we apply the least squares and generalized inverse matrix method, as shown in Equation (5):

(5) PEO=(RBE)1−(RBE)2(RBE)2−(RBE)3(RBE)3−(RBE)4T⋅(RBE)1−(RBE)2(RBE)2−(RBE)3(RBE)3−(RBE)4−1⋅(RBE)1−(RBE)2(RBE)2−(RBE)3(RBE)3−(RBE)4T⋅(PBE)2−(PBE)1(PBE)3−(PBE)2(PBE)4−(PBE)3

The robot’s end-surgical instruments are then moved into four different postures, and the values of PEO are obtained by substituting the corresponding position and pose matrix RBE and PBE into Equation (5).

**Figure 4:**
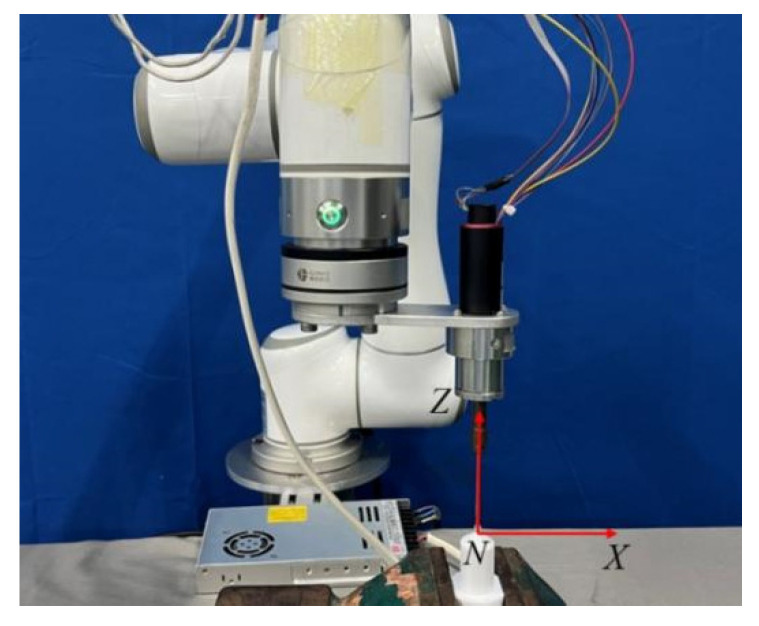
Pose calibration process.

For pose calibration, the “Z/X Direction Method” is utilized. This method involves using the reference point *N* to record the transformation matrix (TBE)n corresponding to this point. Next, the robot end-surgical instrument is moved a specified distance along the *X*-axis in the positive direction, and the transformation matrix (TBE)x is recorded. Then, the instrument is moved a specified distance along the *Z*-axis in the positive direction, and the transformation matrix (TBE)z is recorded. The position and pose calibration process is illustrated in Figure 4.

From Equation (3), we derive the equation presented in Equation (6):

(6) (PBO)i=(RBE)iPEO+(PBE)i

The vector in the positive direction of the *X*-axis of the surgical instrument’s coordinate system at calibration point *N* is determined using the calibration point *N* and the transformation matrix (TBE)x, as shown in Equation (7):

(7) v(x)B=(PBO)x−(PBO)n=(RBE)xPEO−(RBE)nPEO+(PBE)x−(PBE)n

Similarly, the vector in the positive direction of the *Z*-axis is obtained, as shown in Equation (8):

(8) v(z)B=(PBO)z−(PBO)n=(RBE)zPEO−(RBE)nPEO+(PBE)z−(PBE)n

The vector in the positive direction of the *Y*-axis can be determined from the orthogonality between the *X*-axis and *Z*-axis of the surgical instrument’s coordinate system, as shown in Equation (9):

(9) v(y)B=v(z)B×v(x)B

This step ensures the orthogonality of the coordinate system vectors. After calculating the axial vectors for each axis, a normalization operation is performed to derive the spatial position relationship matrix of the surgical instrument relative to the robot flange. This normalized transformation matrix allows for the accurate calibration of the robotic system to replicate the surgeon’s movements during surgery.

(2) Solution of the transformation matrix TOP

**Figure 5:**
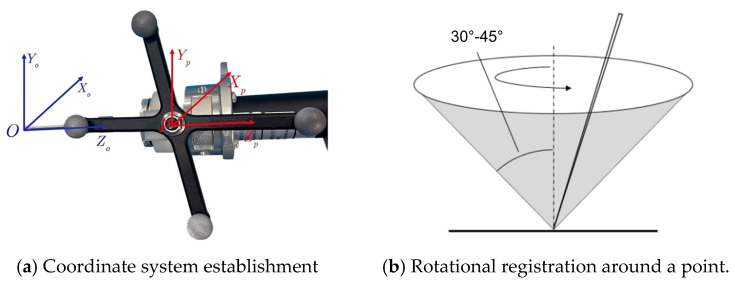
Tip calibration of surgical instruments.

As shown in Figure 5a, the origin of the surgical instrument coordinate system $\left\{O\right\}$ is located at the tip of the spherical milling cutter, while the origin of the optical tracking marker coordinate system $\left\{P\right\}$ is situated at the center of the marker. The coordinate axes of both systems are aligned to ensure consistency, with the only variable being the offset between their positions. Figure 5b illustrates the position calibration process, which is performed using the rotational registration method. In this process, the surgical instrument, equipped with the tracking marker, is rotated around a fixed point at a declination angle between 30° and 45°. During the rotation, data are collected from several distinct position points.

The coordinates of the origin *O* in the coordinate system $\left\{P\right\}$ are denoted as $X={\left(x,y,z\right)}^{T}$ and can be derived using Equation (10):

(10) $$R_{m1}X+T_{m1}=R_{m2}X+T_{m2}=\cdots \cdots R_{mn}X+T_{mn}$$

Using two datasets obtained at different times, Equation (11) is derived as follows:

(11) $$X_{i}={\left(R_{mi}-R_{mi+1}\right)}^{-1}(T_{mi+1}-T_{mi})(i=1,2,3\ldots \ldots ,n-1)$$

The offset X between the two sets of coordinates is averaged and solved as shown in Equation (12):

(12) $$X=\frac{1}{n-1}\sum_{i=1}^{n-1}X_{i}$$

Given that the optical localization system can directly acquire the positional information of the optical tracking markers, the values X for the offsets of the surgical instrument at *n* − 1 positions can be determined. By averaging these offset values, the transformation relationship between the surgical instrument and the optical tracking marker coordinate systems can be established.

This method ensures the precise alignment and positioning of the surgical instruments relative to the optical tracking markers, enhancing the accuracy of the robot’s vertebral plate-cutting operations. By calibrating the offsets and establishing the transformation matrix, the robot can perform highly accurate and reproducible surgical tasks that align with the surgeon’s manual cutting trajectory.

### 2.2. Trajectory Planning for Robotic Vertebral Plate Cutting Based on Surgeon’s Cutting Path

#### 2.2.1. Acquisition and Preprocessing of Point Cloud Data in Robotic Space

**Figure 6:**
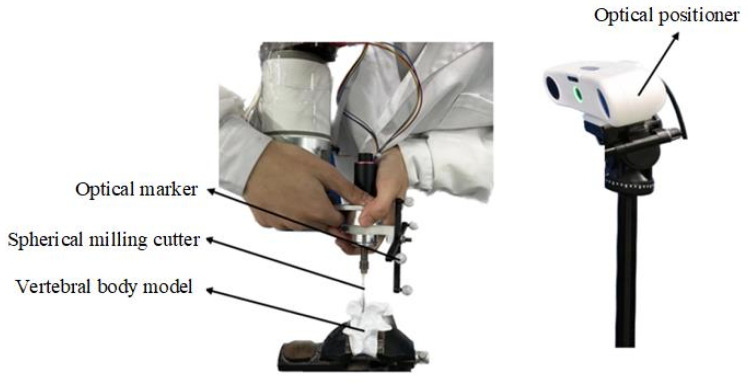
Surgeon cutting the artificial vertebral plate.

To accurately determine the cutting trajectory of the vertebral plate, the robot is initially set to drag mode, and a personalized cutting angle for the artificial vertebral plate is configured. An experienced orthopedic surgeon then operates the surgical instruments mounted on the robot, simulating the layer-by-layer cutting of a 3D-printed artificial vertebral body. The surgeon’s cutting path is recorded using the optical localization system, as shown in Figure 6. During the measurement of the cutting trajectory, it is critical to maintain a constant relative position between the robot base and the optical positioner. Simultaneously, the surgeon must perform the cutting operation based on the personalized path that has been planned for the patient while manipulating the surgical instruments mounted on the robot. Once the cutting data are captured by the optical positioner, a spatial mapping transformation is applied to convert the trajectory information from the optical measurement space to the robotic surgical space.

Due to the challenges in precisely tracking the cutting trajectory and minor errors in the optical positioning system, the collected point cloud data may contain fluctuations and breakpoints, particularly at trajectory inflection points. To improve robotic cutting precision under visibility limitations and environmental interference, we implemented several optimizations: adjusting sensor angles and placement to maximize coverage and avoid obstructions, increasing the number and strategic placement of visual markers on the tool for reliable tracking, and optimizing lighting conditions to reduce interference by using local shadowing, dedicated surgical lighting, and fine-tuning the optical system’s exposure and gain settings.

Given the challenges in controlling the cutting trajectory with high precision and the potential minor errors in the optical positioning system, the point cloud data may exhibit fluctuations and breakpoints, particularly at inflection points of the trajectory, where redundant points may arise. Consequently, preprocessing of the collected point cloud data are necessary, involving steps such as data streamlining and noise reduction. The following outlines the preprocessing steps:

(1) Data streamlining

Based on the data and spatial extent of the original 3D point cloud acquired from vertebral cutting, a cube grid with an appropriate dimension is constructed. The point cloud set $P={\left\{p_{i}\right\}}_{i=1}^{N}$($p_{i}=\in R^{3}$) is partitioned into *n* 3D spatial cubes using the predefined voxel grid edge length L. The center of gravity $V_{i}=(x_{i},y_{i},z_{i})$ of the point set $P_{ij}={\left\{p_{i}\right\}}_{j=1}^{m}(i=1,2,3,\cdots ,n)$ within each voxel cube is computed according to Equation (13). This process identifies all the relevant points within the voxel grid and eliminates redundant cube grids to finalize the simplification of the point cloud data.

(13) $$V_{i}=\frac{1}{m}\sum_{i=1}^{m}p_{i}$$

(2) Noise reduction

A KD-Tree topology is constructed for the streamlined 3D point cloud data to efficiently locate neighboring points. For any point $p_{i}$ in the point set P, k neighboring points are identified. The coordinates of all the neighboring points are retrieved, and their distances $d_{ij}$ from the point $p_{i}$ are calculated. The average distance $d_{i}$ from $p_{i}$ to all its neighbors is then computed as shown in Equation (14):

(14) $$d_{i}=\frac{\sum_{i=1}^{k}d_{ij}}{k-1}$$

The average distance $d_{nj}$ for each point in the point set P is computed, and the standard deviation Std of these distances is derived, as shown in Equation (15):

(15) $$Std=\sqrt{\frac{\sum_{i=1}^{n}{\left(d_{nj}-d_{i}\right)}^{2}}{n-1}}$$

A threshold *T* is established to detect noise points. If the distance between a point $p_{i}$ and all the neighboring points exceeds this threshold, the point is considered noise and is removed. The judgment method of the threshold T is shown in Equation (16), where ω denotes the standard deviation multiple of Std.

(16) $$T=d_{nj}+\omega \times Std$$

By following these preprocessing steps—streamlining and noise reduction—the point cloud data are refined into a cleaner and more accurate representation of the surgeon’s cutting path. This is crucial for ensuring the precise execution of robotic vertebral plate cutting.

#### 2.2.2. Cutting Point Cloud Fitting Based on NURBS Curves

Due to the positioning accuracy of the robotic cutting experimental platform and the jitter generated during the vertebral cutting process, minor fluctuations in the trajectory, particularly in the X−Y−Z directions, may be present in the measured data. To improve the quality of the point cloud trajectory and generate a smoother cutting path, a NURBS curve fitting is employed. The NURBS fitting formula is expressed as follows:

(17) $$p(u)=\frac{\sum_{i=0}^{n}\omega_{i}d_{i}N_{i,k}(u)}{\sum_{i=0}^{n}\omega_{i}N_{i,k}(u)}$$

In Equation (17), n is the number of control vertices, $\omega_{i}$ is the weight factor of the control vertices, $d_{i}$ is the control vertex, u denotes the node of the curve, and $N_{i,k}(u)$ is the *k*-times B-spline basis function on the non-uniform node vector U.

The B-spline basis function $N_{i,k}(u)$ is computed as shown in Equation (18):

(18) $$\left\{\begin{array}{c} N_{i,0}=\left\{\begin{array}{ll} 1 & u\in \left[u_{i},u_{i+1}\right]\\ 0 & \mathrm{else} \end{array}\right.\\ N_{i,k}(u)=\frac{u-u_{i}}{u_{i+k+1}-u}N_{i,k-1}(u)+\frac{u_{i+k}-u}{u_{i+k}-u_{i+1}}N_{i+1,k-1}(u)\\ \frac{0}{0}=0 \end{array}\right.$$

In Equation (18), $U=[u_{0},u_{1},\ldots ,u_{n+k+1}]$ represents the node vector and k is the number of spline bases. The B-spline basis function recursively combines lower-degree basis functions to construct the higher-order curve.

When fitting the NURBS curves, parameters such as node vectors, control vertices, and weight factors must be determined. Since the optimization of weight factors for NURBS curve fitting is still in its early stages, the weight factor is set to 1, and only the optimization of the node vectors and control vertices is considered. Given a set of processed point cloud data, represented as type-valued points $p_{i}(x_{i},y_{i},z_{i})$, the node vectors and control vertices are computed as follows:

In this study, third-degree NURBS curves are fitted, with node vector subscripts of n+6. To generate smooth and accurate curves, the node vectors are computed using the cumulative chord length parameterization method, as shown in Equation (19):

(19) $$\left\{\begin{array}{c} u_{0}=u_{1}=u_{3}=u_{4}=0\\ u_{n+3}=u_{n+4}=u_{n+5}=u_{n+6}=1\\ u_{i+3}=u_{i+4}+△p_{i},i=1,2,\ldots ,n-1 \end{array}\right.$$

In the above equation, $△p_{i}=\frac{p_{i}-p_{i-1}}{s}$ represents the forward differential vector.

The computational procedure for representing the control vertices in matrix form, using the cut-loss boundary condition, is shown in Equation (20):

(20) $$\left[\begin{array}{ccccccc} 1 & & & & & & \\ a_{2} & b_{2} & c_{2} & & & & \\ & . & . & . & & & \\ & & . & . & . & & \\ & & & . & . & . & \\ & & & & a_{n} & b_{n} & c_{n}\\ & & & & & & 1 \end{array}\right]\left[\begin{array}{c} d_{1}\\ d_{2}\\ .\\ .\\ .\\ d_{n}\\ d_{n+1} \end{array}\right]=\left[\begin{array}{c} e_{1}\\ e_{2}\\ .\\ .\\ .\\ e_{n}\\ e_{n+1} \end{array}\right]$$

In the above equation, $d_{i}$ represents the control vertex, and let $\Delta_{i}=u_{i+1}-u_{i}(i=0,1,\ldots ,n)$, then we obtain Equations (21) and (22):

(21) $$\left\{\begin{array}{c} a_{i}=\frac{{\left(\Delta_{i+2}\right)}^{2}}{\Delta_{i}+\Delta_{i+1}+\Delta_{i+2}}\\ b_{i}=\frac{\Delta_{i+2}(\Delta_{i}+\Delta_{i+1})}{\Delta_{i}+\Delta_{i+1}+\Delta_{i+2}}+\frac{\Delta_{i+1}(\Delta_{i+2}+\Delta_{i+3})}{\Delta_{i+1}+\Delta_{i+2}+\Delta_{i+3}}\\ c_{i}=\frac{{\left(\Delta_{i+1}\right)}^{2}}{\Delta_{i+1}+\Delta_{i+2}+\Delta_{i+3}} \end{array}\right.$$

(22) $$\left\{\begin{array}{c} e_{1}=p_{0}-\frac{\Delta_{3}}{3}p_{0}\\ e_{i}=(\Delta_{i+1}+\Delta_{i+2})p_{i-1}\\ e_{n+1}=p_{n}-\frac{\Delta_{n+2}}{3}p_{n} \end{array}\right.$$

Finally, once the NURBS curve is fitted using the above steps, the curve is discretized using MATLAB’s toolbox to obtain the required robot cutting point cloud coordinates for generating the subsequent robotic cutting trajectory.

These procedures ensure the creation of a high-quality, smooth, and accurate cutting trajectory, which is essential for improving the robotic vertebral plate-cutting process based on the surgeon’s preoperative simulation. By fitting the surgeon’s cutting path with NURBS curves, we can enhance the precision and consistency of the robot’s cutting behavior, leading to more reliable and effective surgical outcomes.

#### 2.2.3. Robotic Vertebral Plate-Cutting Trajectory Generation

**Figure 7:**
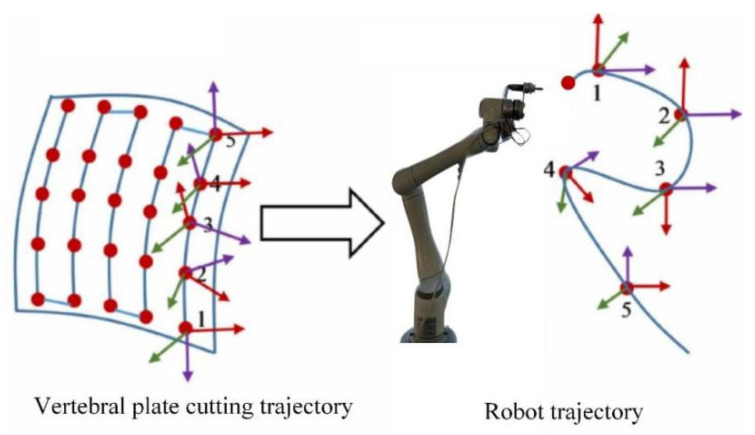
Transformation of robot motion trajectory, where point 1–5 are just schematic representations of trajectory points.

After completing the spatial mapping transformation, preprocessing, trajectory fitting, and solving for the discrete point cloud data obtained from the vertebral plate-cutting procedure, the cutting trajectory for the 3D-printed artificial vertebral plate in the robot’s workspace is derived. To enable the robotic cutting operation, however, this trajectory information must be translated into the robot’s motion trajectory, as depicted in Figure 7.

The method for generating the robotic cutting trajectory involves the following steps:

(1) Point selection and definition

A point cloud set P is selected from the robotic cutting point cloud data. Any point $p_{ij}$ within this set P is chosen as the current cutting point. We denote the position vector of this point as p, and the normal vector at this point as $v_{n}$. The direction from the current cutting point to the next cutting point is designated as the tangent vector $v_{t}$, which represents the direction in which the spherical milling cutter moves. This tangent vector guides the robot’s tool path in the subsequent cutting operation.

(2) Local coordinate system establishment

A cutting point coordinate system, denoted as $\left\{p_{ij}\right\}$, is established with the current cutting point $p_{ij}$ as the origin. The coordinate axes are defined using $v_{n}$ as the primary axis, $v_{t}$ as the secondary axis, and the binormal vector $v_{e}$, which is obtained by the cross product of $v_{n}$ and $v_{t}$. The cutting point coordinate system is then transformed into the robot base coordinate system using a matrix transformation, which ensures compatibility with the robot’s motion model.

(3) Transformation matrix calculation

The transformation matrix TpijB that converts the cutting point coordinate system into the robot base coordinate system is calculated as shown in Equation (23):

(23) TpijB=vtvevnp0001

This matrix represents the relationship between the local coordinate system at the cutting point and the robot’s base coordinate system, providing the necessary transformation for the robot to follow the cutting trajectory accurately.

(4) Robot position matrix

The position matrix TEpijB for the robot describes the robot’s joint configuration in relation to the cutting point. The robot’s motion trajectory is determined through inverse kinematics, which allows the robot to achieve the desired cutting path by adjusting its joint angles to match the calculated trajectory. The robot’s position matrix is expressed in Equation (24):

(24) TEpijB=TpijBTEO

In Equation (25), the inverse kinematic model of the robot is used to calculate the required joint angles $\Theta (\theta_{1},\theta_{2},\ldots ,\theta_{6})$:

(25) Θ=Γ−1(TEpB)

Here, $\Gamma^{-1}$ represents the inverse kinematic function that transforms the cutting trajectory information from the tool frame (cutting point coordinate system) into the corresponding robot joint angles required to follow the specified cutting trajectory. By solving this system, the robot can replicate the surgeon’s planned cutting path, ensuring precision in the vertebral plate-cutting operation.

These steps collectively enable the transformation of the surgeon’s cutting path into a robotic motion trajectory, guiding the robot’s movements with high accuracy and ensuring that the cutting procedure follows the surgeon’s intended trajectory, thus improving the quality and precision of robotic vertebral plate cutting.

### 2.3. BP Neural Network-Based Trajectory Parameter Prediction

#### 2.3.1. Establishment of BP Neural Network Prediction Model

The BP neural network prediction model is utilized to forecast cutting trajectory parameters based on the specified cutting region by an experienced surgeon. This model aims to generate a personalized cutting trajectory for each patient, thereby reducing preoperative planning time and enhancing surgical safety.

(1) Cutting region determination

**Figure 8:**
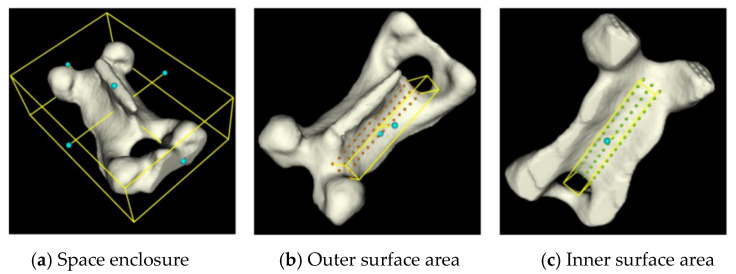
Three-dimensional image for determining the vertebral plate-cutting area.

Before performing vertebral plate cutting, the surgeon must evaluate the type and location of the lesion, and define the cutting area using preoperative medical imaging techniques. A high-quality vertebral bone model is constructed using 3D image reconstruction technology, and an interactive spatial envelope box is created. This box approximates the cutting area as a rectangular body, as shown in Figure 8.

(2) Cutting trajectory characteristics

**Figure 9:**
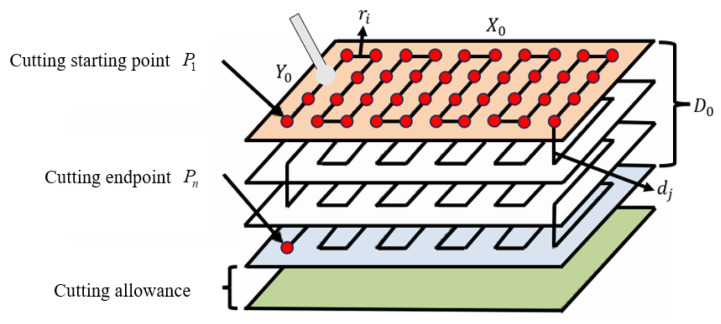
Parameters of the vertebral plate-cutting trajectory.

The cutting trajectory begins at the outer surface corner of the vertebral plate. Using a layer-by-layer cutting strategy, each cutting layer’s start point is connected to the end of the previous layer. The direction alternates between neighboring layers to form a complete cutting trajectory. Figure 9 illustrates this cutting trajectory, where parameters such as cutting length, width, depth, row spacing, and cut depth are defined. In the figure, $P_{i}$ represents the boundary vertex of the cutting region of the vertebral plate, $Y_{0}$ denotes the cutting length, $X_{0}$ indicates the cutting width, $D_{0}$ specifies the cutting depth, $r_{i}$ refers to the row spacing of the cutting trajectory, and $d_{j}$ represents the depth of a single cut. These parameters characterize the cutting region of the vertebral plate and are personalized for each patient, enabling the determination of the robot’s personalized cutting trajectory, including the number of horizontal cutting row spacing h and the number of layers for the longitudinal cutting depth c.

(3) BP neural network architecture

The BP neural network used in this study consists of one input layer, one hidden layer, and one output layer to ensure accurate model predictions. The input layer contains two neurons, representing the width and depth of the cutting area on the vertebral plate. These inputs are normalized before being fed into the network. The output layer also contains two neurons, representing the number of horizontal cutting trajectory row spacings and the number of vertical cutting depth layers, which are subject to inverse normalization.

**Figure 10:**
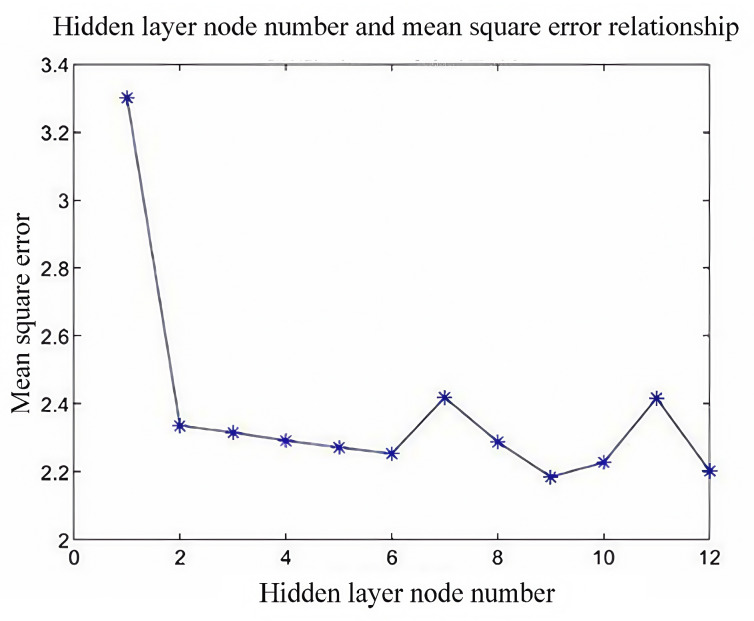
Determination of the optimal number of nodes for the implicit layer.

The number of hidden neurons is determined using the empirical formula in Equation (26), where both the input and output layers contain 2 neurons. The experimental results (Figure 10) indicate that the mean square error (MSE) is minimized with 9 hidden nodes, making this the optimal number.

(26) $$\left\{\begin{array}{c} k=\sqrt{n+m}+(1\sim 10)\\ k=\log_{2}n \end{array}\right.$$

In the above equation, n and m denote the number of neurons in the input and output layers, respectively, with input layer n = 2 and output layer m = 2.

(4) Training the neural network

**Figure 11:**
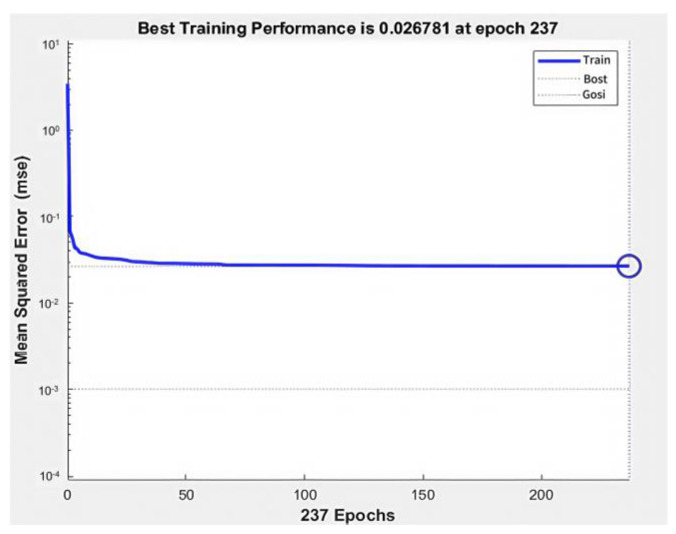
Network training using combinations of transfer functions in Group 6.

The training was conducted using six transfer function combinations between layers, as shown in Table 1. The combinations were tested for convergence speed and training error. Figure 11 shows that the combination of the tansig function between the input and hidden layers and the purelin function between the hidden and output layers achieved the fastest convergence and lowest error. Thus, this combination was chosen for the neural network.

In addition to selecting the transfer function, the appropriate training function must also be chosen. Common training functions include Levenberg–Marquardt (trainlm), resilient backpropagation (trainrp), and gradient descent (traingd). In this study, the Levenberg–Marquardt algorithm (trainlm) is determined to be the most suitable for training the neural network.

(5) Performance evaluation

The BP neural network model utilizes two input parameters—the width $X_{0}$ and depth $D_{0}$ of the vertebral plate-cutting area—and produces two output parameters: the number of horizontal cutting rows h and the number of vertical cutting depth layers c. The model aims to establish a mapping relationship between the input and output parameters, and its performance is evaluated through the metrics in Table 2.

Due to the inherent variability in vertebral plate-cutting areas, which can be influenced by factors such as age, gender, and disease duration, the acquisition of a sufficient and representative sample dataset is essential. To this end, 3D models were generated using vertebral body data provided by partner hospitals, allowing for the precise determination of cutting areas and trajectory parameters. A total of 118 personalized datasets were collected, comprising 68 male and 50 female patients, aged between 42 and 73 years (with a mean age of 57.6 ± 14.9 years), and with disease durations ranging from 10 to 31 months (mean duration: 17.5 ± 6.3 months). Detailed input and output parameters are presented in Table 2.

**Figure 12:**
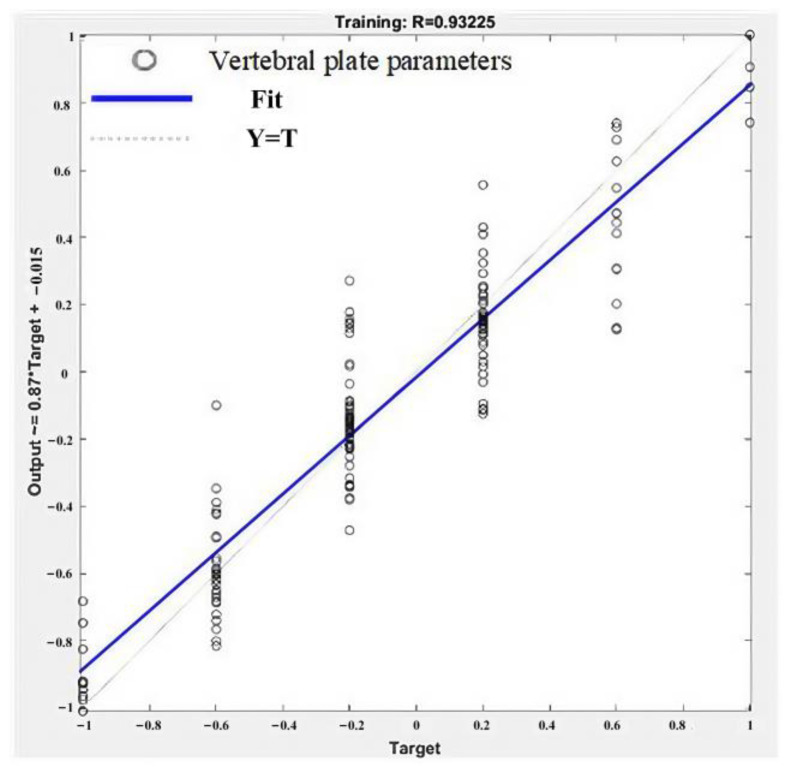
Linear regression plot and corresponding correlation coefficient values.

Following the training of the BP neural network, the performance of the model was evaluated through a linear regression analysis, as shown in Figure 12. The predicted values closely aligned with the actual experimental data, achieving a correlation coefficient of 0.93225, which demonstrates satisfactory training accuracy.

**Figure 13:**
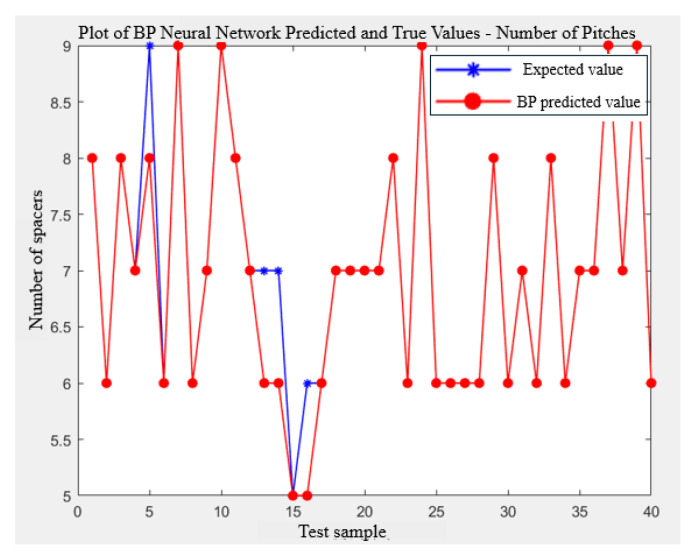
Comparison of the number of rows.

**Figure 14:**
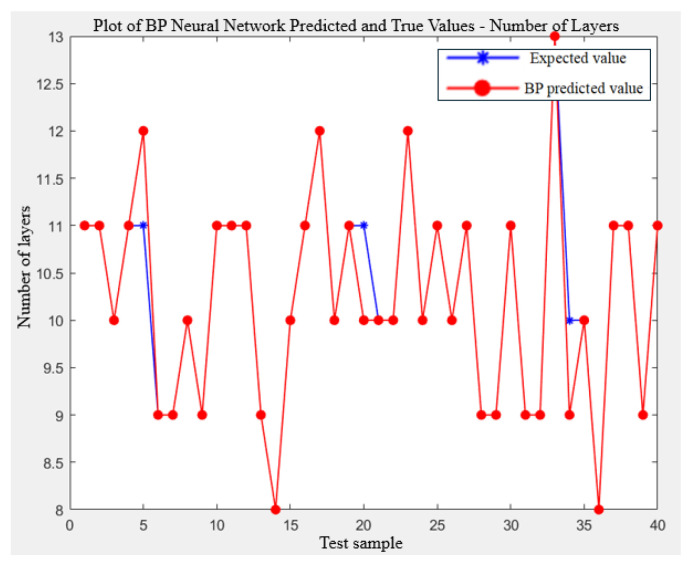
Comparison of the number of layers.

Figure 13 and Figure 14 provide visual comparisons between the predicted and actual values for the number of cutting rows and layers, respectively. These figures show that the model’s predictions are highly accurate across most data points.

The performance evaluation metrics for the prediction model are as follows: for the number of layers, the MSE is 0.075, the root mean square error (RMSE) is 0.27386, and the goodness of fit is 0.94166. For the number of rows, the MSE is 0.1, the RMSE is 0.31623, and the goodness of fit is 0.92812. These results indicate that the BP neural network model demonstrates robust predictive accuracy and reliability in forecasting the trajectory parameters for vertebral plate cutting.

(6) Model Stability

To assess the stability of the BP neural network, 13 reserved sample datasets were used for model verification, and the predicted vs. experimental values are shown in Table 3. Discrepancies were observed in only the 12th sample, confirming the model’s high prediction accuracy and stability. This evaluation demonstrates that the BP neural network model is capable of predicting trajectory parameters with high accuracy and stability, meeting the requirements for personalized robotic vertebral plate cutting.

#### 2.3.2. Robot Trajectory Planning Based on Trajectory Parameters

The BP neural network model has demonstrated its capability in accurately predicting cutting trajectory parameters, specifically the row spacing h and number of layers c for vertebral plate cutting. These parameters are used to calculate the average travel distance $\bar{r}$ and the average depth of cut $\bar{d}$ for a single cut using the formula in Equation (27):

(27) $$\left\{\begin{array}{c} \bar{r}=\frac{X_{0}}{h}\\ \bar{d}=\frac{D_{0}}{c} \end{array}\right.$$

**Figure 15:**
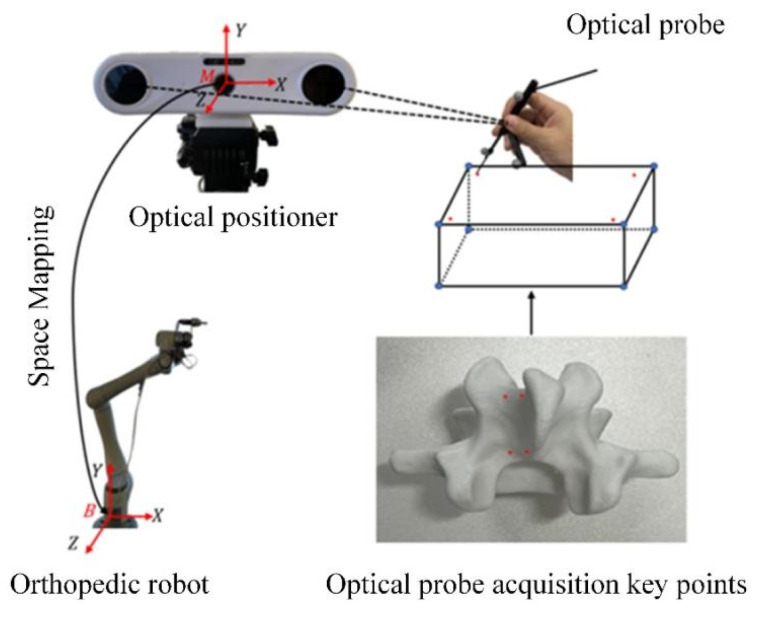
Mapping relationship of probe acquisition points.

After determining these parameters, the next step involves mapping the trajectory coordinates. A 3D-printed personalized vertebral model is placed in the optical measurement space, where an optical probe tool is used to trace the boundaries of the cutting area. These measurements provide the key point data in the optical measurement coordinate system. The data are then transformed into the robot’s coordinate system through the established relationship between the optical localization system and the robot, as depicted in Figure 15.

(1) Cutting trajectory calculation

**Figure 16:**
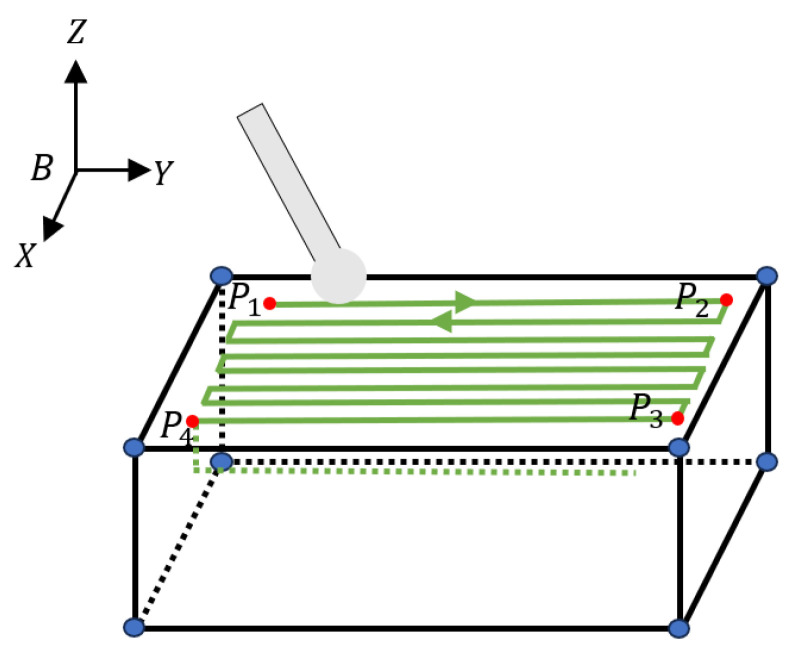
Cutting trajectory diagram.

**Figure 17:**
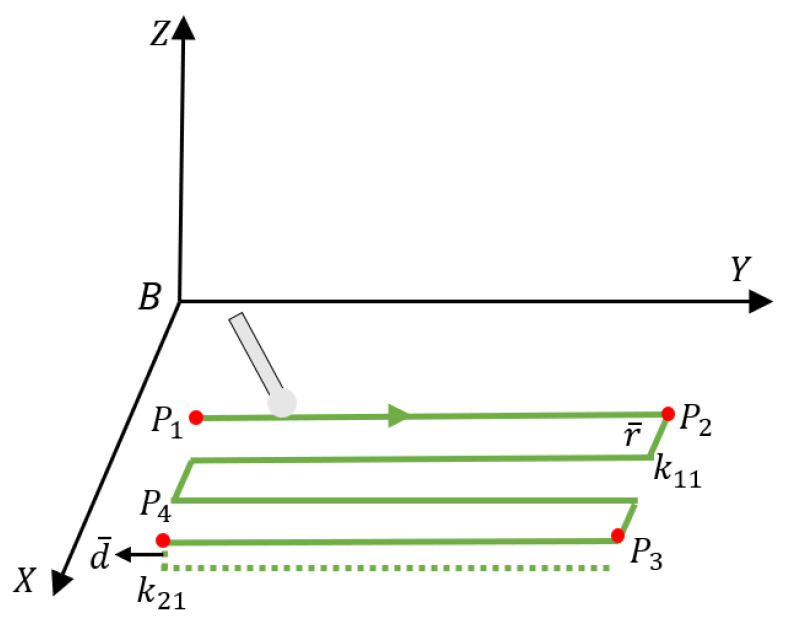
Local trajectory diagram.

To accurately determine the cutting trajectory of the robot, it is essential to identify the inflection points $k_{ij}$ (see Figure 16 and Figure 17), where ij represents the jth inflection point of the ith layer. The coordinates of the inflection points $k_{1j}$ in the first layer are calculated as follows: since all the inflection points lie on vectors $\vec{P_{1}P_{4}}$ and $\vec{P_{2}P_{3}}$, their coordinates $k_{1j}$ can be determined by calculating the lengths of the vectors and the unit vectors. The process is as follows:

Coordinate calculation: Let the coordinates of $P_{1},P_{2},P_{3}$, and $P_{4}$ be ($x_{1},y_{1},z_{1}$), ($x_{2},y_{2},z_{2}$), ($x_{3},y_{3},z_{3}$), and ($x_{4},y_{4},z_{4}$), respectively. Let $k_{11}$ be the first inflection point on vector $\vec{P_{2}P_{3}}$, and the distance between $P_{2}$ and $k_{11}$ be $\bar{r}$. The coordinates of $k_{11}$ are solved using Equation (28):

(28) $$k_{11}=P_{2}+\bar{r}(\vec{P_{2}P_{3}}/\left\|\vec{P_{2}P_{3}}\right\|)$$

In Equation (28), $\left\|\vec{P_{2}P_{3}}\right\|$ denotes the mode of the vector $\vec{P_{2}P_{3}}$. The coordinate values of the inflection point $k_{12}$ are solved by Equation (29):

(29) $$k_{12}=P_{1}+\bar{r}(\vec{P_{1}P_{4}}/\left\|\vec{P_{1}P_{4}}\right\|)$$

Depth offset for subsequent layers: Coordinates for the other inflection points $k_{1j}$ are calculated similarly. The cutting depth $\bar{d}$ is used to offset the subsequent layer of vertebral cutting; specifically, the offset along the direction of the normal vector to the cutting surface is $\bar{d}$ mm. The coordinates after the offset can be expressed using Equation (30):

(30) $$\left\{\begin{array}{c} k_{2j}=k_{1j}-(\vec{e_{n}}\times \bar{d})\\ {P_{m}}^{2}=P_{m}-(\vec{e_{n}}\times \bar{d}),(m=1,2,3,4) \end{array}\right.$$

where $k_{2j}$ are the second layer inflection coordinates, ${P_{m}}^{2}$ are the second layer vertex coordinates, and $\vec{e_{n}}$ represents the unit normal vector of the cutting surface. Following the above method, the coordinates of all the inflection points and vertices can be computed in turn.

(2) Corner rounding algorithm

**Figure 18:**
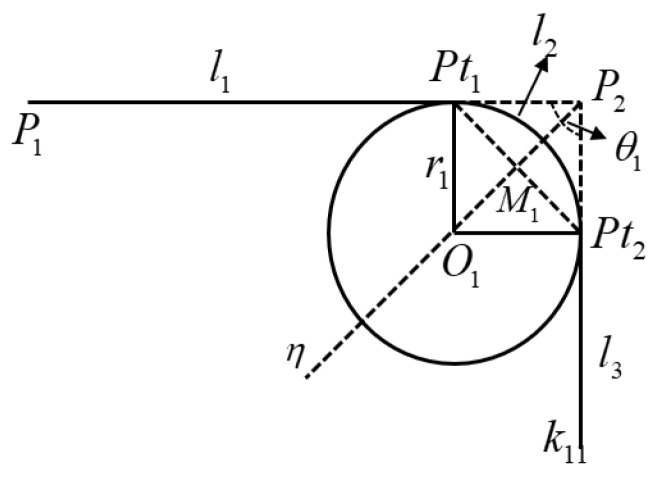
Analysis of cutting trajectory corner rounding.

Once the coordinates of the corner positions are obtained, it is crucial to round these corners to minimize vibrations and wear during the cutting process. For instance, the first corner in Figure 17 undergoes a corner rounding process, as shown in Figure 18.

Figure 18 depicts the analysis of the cutting trajectory corner rounding, where $O_{i}$ and $r_{j}$ are the center and radius of the transition arc, $Pt_{i}$ is the transition point between the straight line and the arc, and $M_{i}$ is the midpoint of the transition point. $l_{i}$, $\theta_{i}$, and η denote the length of the segmented path, the path turn angle, and the angle bisector of the turn angle, respectively.

The transition arc helps smooth the path between sharp turns, minimizing friction and vibration. The calculations involved in this process are as follows:

Turning angle calculation: The turning angle $\theta_{1}$ at each corner is calculated using Equation (31):

(31) $$\theta_{1}=\arccos\frac{\vec{P_{1}P_{2}}\cdot \vec{P_{2}k_{11}}}{\left|\vec{P_{1}P_{2}}\right|\left|\vec{P_{2}k_{11}}\right|}$$

Radius calculation: The radius $r_{1}$ of the transition arc by transition points $Pt_{1}$ and $Pt_{2}$ is determined using Equation (32):

(32) $$r_{1}=\left\{\begin{array}{c} s_{1}\left|\vec{P_{1}P_{2}}\right|\tan\frac{\theta_{1}}{2},\left|\vec{P_{1}P_{2}}\right|\leq \left|\vec{P_{2}k_{11}}\right|\\ s_{1}\left|\vec{P_{2}k_{11}}\right|\tan\frac{\theta_{1}}{2},\left|\vec{P_{1}P_{2}}\right|\geq \left|\vec{P_{2}k_{11}}\right| \end{array}\right.$$

(33) $$\vec{P_{2}P_{t1}}=\frac{r_{1}/\tan\frac{\theta_{1}}{2}}{\left|\vec{P_{1}P_{2}}\right|}\vec{P_{1}P_{2}},\;\vec{P_{2}P_{t2}}=\frac{r_{1}/\tan\frac{\theta_{1}}{2}}{\left|\vec{P_{2}k_{11}}\right|}\vec{P_{2}k_{11}}$$

In Equation (32), $s_{1}$ is the arc radius adjustment parameter, which can be set according to the specific trajectory.

Center coordinate calculation: The coordinates of the arc center $O_{1}$ and $M_{1}$ are calculated using Equation (34):

(34) $$\left\{\begin{array}{c} \vec{P_{t1}M_{1}}=\frac{\vec{P_{t1}P_{t2}}}{2}\\ \vec{P_{2}O_{1}}=\frac{\left|\vec{P_{2}O_{1}}\right|}{\left|\vec{P_{2}M_{1}}\right|}\vec{P_{2}M_{1}}=\frac{r_{1}}{\sin(\theta_{1}/2)} \end{array}\right.$$

By applying these corrections, the robot’s cutting trajectory is smoothly adjusted, reducing vibration and enhancing the precision of the cutting process.

Robot trajectory planning for vertebral plate cutting involves accurately determining the key position parameters using BP neural network predictions. These parameters, such as the number of rows and layers, along with cutting depths, are used to compute the cutting trajectory. The procedure also incorporates adjustments for smooth transitions at corner points, utilizing a rounding algorithm that enhances the cutting process’s efficiency and precision.

### 2.4. Registration of Artificial Vertebra to Patient Vertebra

**Figure 19:**
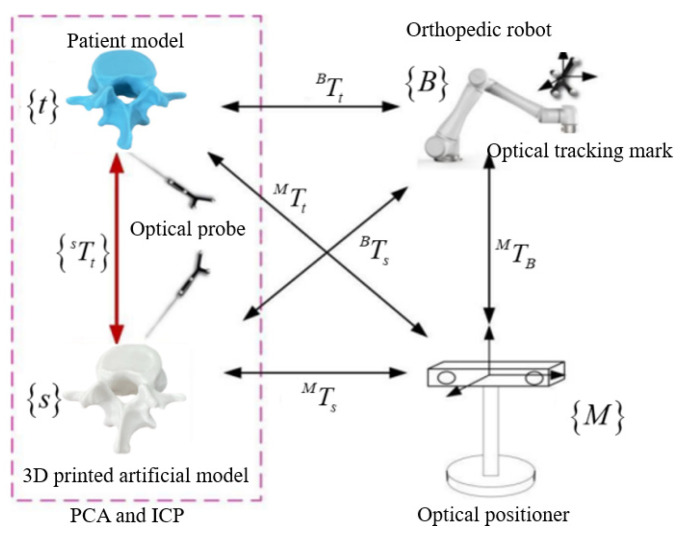
Registration of artificial vertebra to patient vertebra.

To apply the preoperative cutting trajectory to an actual patient’s vertebral decompression surgery, a key step is establishing the registration relationship between the 3D-printed artificial vertebral body and the patient’s vertebral body. This is illustrated in Figure 19, where the optical positioning system’s probe tool is used to capture the characteristic point cloud information of the 3D-printed artificial vertebral body. A point cloud registration algorithm is then employed to align the 3D-printed model with the patient’s actual vertebra, ensuring that the robot can replicate the surgeon’s cutting trajectory accurately.

In this study, the Principal Component Analysis (PCA) and Iterative Closest Point (ICP) algorithms are utilized to perform coarse and fine registration of the point cloud, respectively, to finalize the registration relationship. The registration process is as follows:

(1) Construct the point cloud datasets for the artificial and patient vertebrae, as shown in Equations (35) and (36):
  (35) $$p_{s}=\left[\begin{array}{ccc} x_{1} & y_{1} & z_{1}\\ x_{2} & y_{2} & z_{2}\\ \vdots & \vdots & \vdots \\ x_{n} & y_{n} & z_{n} \end{array}\right]$$
  (36) $$p_{t}=\left[\begin{array}{ccc} x_{1}^{\prime } & y_{1}^{\prime } & z_{1}^{\prime }\\ x_{2}^{\prime } & y_{2}^{\prime } & z_{2}^{\prime }\\ \vdots & \vdots & \vdots \\ x_{n}^{\prime } & y_{n}^{\prime } & z_{n}^{\prime } \end{array}\right]$$
(2) Calculate the centers of mass $\mu_{s}$ and $\mu_{t}$ of the two point cloud sets, as shown in Equation (37):
  (37) $$\left\{\begin{array}{c} \mu_{s}=\frac{1}{n}\sum_{i=1}^{n}p_{s_{i}}\\ \mu_{t}=\frac{1}{m}\sum_{i=1}^{m}p_{t_{i}} \end{array}\right.$$
(3) Determine the covariance matrix for both point cloud sets:

For the artificial vertebral point cloud dataset, as shown in Equation (38):

(38) $$cov_{s}\left[\begin{array}{ccc} cov(X,X) & cov(X,Y) & cov(X,Z)\\ cov(Y,X) & cov(Y,Y) & cov(Y,Z)\\ cov(Z,X) & cov(Z,Y) & cov(Z,Z) \end{array}\right]$$

For the patient vertebral point cloud dataset, as shown in Equation (39):

(39) $$cov_{t}\left[\begin{array}{ccc} cov(X^{\prime },X^{\prime }) & cov(X^{\prime },Y^{\prime }) & cov(X^{\prime },Z^{\prime })\\ cov(Y^{\prime },X^{\prime }) & cov(Y^{\prime },Y^{\prime }) & cov(Y^{\prime },Z^{\prime })\\ cov(Z^{\prime },X^{\prime }) & cov(Z^{\prime },Y^{\prime }) & cov(Z^{\prime },Z^{\prime }) \end{array}\right]$$

Here, X, Y, and Z with $X^{\prime }$, $Y^{\prime }$, and $Z^{\prime }$ denote the 3 column vectors of the artificial vertebral point set matrix and the patient vertebral point set matrix, respectively.

(4) Solve the covariance matrix eigenvectors and eigenvalues of the two point clouds in MATLAB, sorting the eigenvalues in descending order to obtain the eigenvector matrices $T_{s}$ and $T_{t}$, as shown in Equation (40):
  (40) $$T_{t}=T_{s}\times (T\times R)$$

In Equation (40), T is the translation matrix between $T_{s}$ and $T_{t}$, and R is the rotation matrix between $T_{s}$ and $T_{t}$. Assuming any point $s_{i}$ in the artificial vertebral point cloud set $p_{s}$, the point $s_{i}^{\prime }$ denotes the point $s_{i}$ transformed to the point under the spatial coordinate system where the patient’s vertebral point cloud set $p_{t}$ is located, as shown in Equation (41):

(41) $$s_{i}^{\prime }=s_{i}\times (T\times R)$$

(5) Achieve the coarse registration of the artificial vertebral point cloud set with the patient vertebral point cloud set through the aforementioned steps. Next, perform the fine registration of the point cloud using the ICP algorithm:

Let $p_{s}$ and $p_{t}$ denote the artificial vertebral point cloud set and the patient vertebral point cloud set, respectively, represented by Equations (42) and (43):

(42) $$p_{s}=\left\{s_{i}\right\}\;i=1,2,3,\cdots ,n$$

(43) $$p_{t}=\left\{t_{i}\right\}\;i=1,2,3,\cdots ,m$$

Use the unit quaternion method to denote the rotational transformation vector $q_{r}={\left[q_{0},q_{1},q_{2},q_{3}\right]}^{T}$ ($q_{0}\geq 0$). The rotational transformation matrix is $R(q_{r})$, and the translation transformation vector is $q_{t}={\left[q_{4},q_{5},q_{6}\right]}^{T}$. The problem of solving the sum of the minimum Euclidean distances of the corresponding points can be transformed into solving $q_{r}$ and $q_{t}$, minimizing $f(q_{t},q_{r})$, where $f(q_{t},q_{r})$ is expressed in Equation (44):

(44) $$f(q_{t},q_{r})=\frac{1}{n}{\sum_{i=1}^{n}\left\|t_{i}-R(q_{r})s_{i}-q_{t}\right\|}^{2}$$

(6) Calculate the centers of gravity $s_{o}$ and $t_{o}$ for the artificial and patient vertebrae point cloud sets, respectively, as shown in Equations (45) and (46):
  (45) $$s_{o}=\frac{1}{n}\sum_{i=1}^{n}s_{i}$$
  (46) $$t_{o}=\frac{1}{m}\sum_{i=1}^{m}t_{i}$$
(7) Construct the covariance matrix from the two points sets $p_{s}$ and $p_{t}$, resulting in a symmetric matrix as shown in Equation (47):
  (47) $$Q(\sum_{t,s})=\left[\begin{array}{cc} tr(\sum_{t,s}) & \Delta^{T}\\ \Delta & \sum_{t,s}+\sum_{t,s}^{T}-tr(\sum_{t,s})I_{3} \end{array}\right]$$

Here, $I_{3}$ is a 3 × 3 unit matrix, $tr(\sum_{t,s})$ denotes the trace of the matrix $\Delta ={\left[A_{23}\;A_{31}\;A_{12}\right]}^{T}$, and $A_{i,j}$ is the antisymmetric matrix of the matrix $A_{i,j}=(\sum_{t,s}-\sum_{t,s}^{T}{)}_{i,j}$.

(8) Calculate the eigenvectors and eigenvalues of $Q(\sum_{t,s})$, where the eigenvector corresponding to the largest eigenvalue is the optimal rotation vector $q_{r}$, and the expression of $q_{r}$ is shown in Equation (48):
  (48) $$R(q_{r})=\left[\begin{array}{ccc} q_{0}^{2}+q_{1}^{2}-q_{2}^{2}-q_{3}^{2} & 2(q_{1}q_{2}-q_{0}q_{3}) & 2(q_{1}q_{3}+q_{0}q_{2})\\ 2(q_{1}q_{2}+q_{0}q_{3}) & q_{0}^{2}-q_{1}^{2}+q_{2}^{2}-q_{3}^{2} & 2(q_{2}q_{3}-q_{0}q_{1})\\ 2(q_{1}q_{3}-q_{0}q_{2}) & 2(q_{2}q_{3}+q_{0}q_{1}) & q_{0}^{2}-q_{1}^{2}-q_{2}^{2}+q_{3}^{2} \end{array}\right]$$
(9) Calculate the optimal translation vector $q_{t}$ according to Equation (49):
  (49) $$q_{t}=t_{o}-R(q_{r})s_{o}$$
(10) After completing the registration, analyze the accuracy using the RMSE as shown in Equation (50):
  (50) $$RMSE=\sqrt{\frac{{\sum_{i=1}^{n}\left\|p_{t_{i}}-(q_{r}\cdot p_{s_{i}}+q_{t})\right\|}^{2}}{n}}$$

By using the PCA and ICP algorithms for coarse and fine registration, the artificial vertebra is accurately aligned with the patient’s vertebra. The transformation parameters—rotation and translation—are computed through a series of matrix manipulations and eigenvalue calculations. This process ensures the precise mapping of the preoperative cutting trajectory to the patient’s vertebral anatomy, enabling accurate robotic surgery based on the preoperative plan.

## 3. Results and Discussion

### 3.1. Accuracy Validation of Spatial Mapping Relationships for Robotic Systems

The accuracy of the robot’s cutting trajectory, which replicates the surgeon’s movements, is critically dependent on the precision of the spatial mapping relationship between the robot and the optical measurement system. To ensure this accuracy, verification experiments were conducted, during which the relative positions of the optical positioner and the robot remained unchanged. Surgical instruments equipped with optical tracking markers were moved to various positions, and the optical positioning system, utilizing AimToolBoxV2.3.3, collected and processed real-time position information.

(1) Experimental setup and data collection

The experiments were carried out in six sets, with each set involving the collection of six calibration points through the optical localization system. The coordinates of these calibration points, measured by the optical positioning system, are provided in Table 4.

Using the spatial mapping relationship, the coordinates of the calibrated points within the robot’s base coordinate system were computed using the transformation relationship between coordinate systems, as detailed in Table 5. After multiple coordinate transformation experiments, the transformation matrix TBM between the robot’s base coordinate system and the optical measurement coordinate system was obtained.

(2) Verification of spatial mapping accuracy

To further verify the spatial mapping accuracy, 10 sets of verification points were collected under the optical localization system. The coordinates of these points, representing the target positions for the robot’s end effector, were transformed into the robot’s coordinate system using the derived spatial mapping transformation. The robot was then directed to these target positions, and the actual position achieved by the robot was recorded using the optical tracking markers. The deviation between the target and actual positions was computed using the Euclidean distance algorithm. Table 6 summarizes the results of this accuracy verification, showing the deviation (in mm) between the target and actual positions for each of the 10 datasets.

(3) Position deviation analysis

The data in Table 6 indicate that the average deviation between the target and actual positions is approximately 0.558 mm, which reflects a high degree of accuracy. The deviation remains relatively stable across the 10 datasets, though minor fluctuations are observed. These fluctuations are likely due to the multiple conversion steps involved in deriving the spatial mapping relationship, which can introduce small cumulative errors.

**Figure 20:**
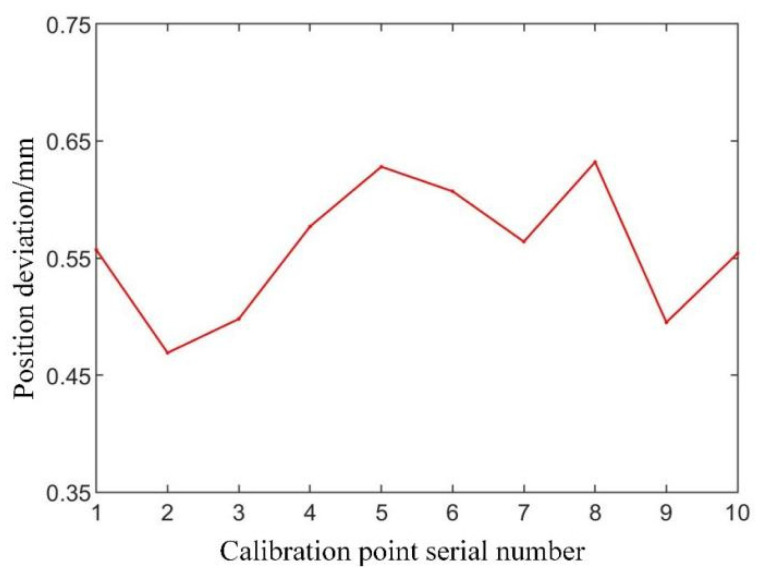
Positional deviation in the spatial mapping relationship.

Figure 20 illustrates the positional deviation across the 10 verification datasets. From the figure, it can be seen that the deviation fluctuates slightly but remains within an acceptable range, which suggests that the mapping relationship is stable and reliable.

This analysis shows that the spatial mapping relationship used for the robotic system is accurate and reliable, ensuring that the robot can replicate the surgeon’s cutting trajectory with a minimal deviation, which is critical for precise surgical operations.

### 3.2. Verification of Trajectory Planning Simulation Under Surgeon’s Cutting Trajectory

**Figure 21:**
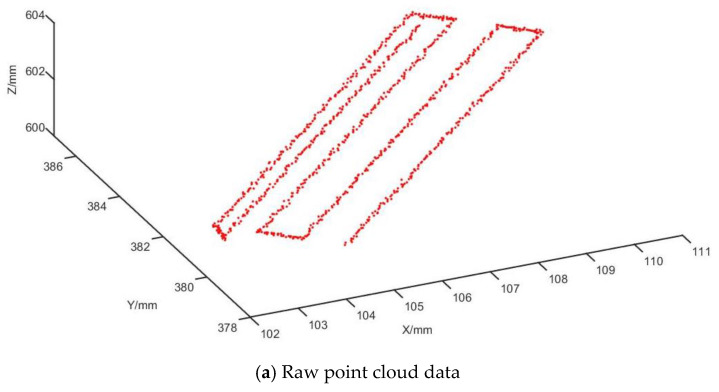

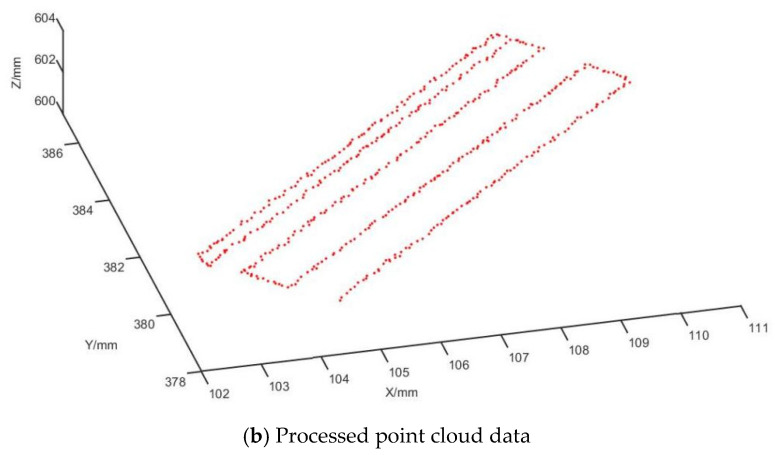
Raw point cloud data and post-filtering point cloud data.

To validate the accuracy of the trajectory planning and ensure it replicates the surgeon’s cutting trajectory, a personalized vertebral body model was created using 3D printing based on the patient’s spinal CT data. This model was then used to guide the robot during the cutting experiment. The surgical plan and cutting path were designed by the surgeon, who operated the robot to perform the cutting procedure on the artificial vertebrae. The raw trajectory point cloud data were recorded using an optical positioner, as shown in Figure 21a. The original point cloud data were preprocessed to enhance the accuracy and stability of the robot’s trajectory, as depicted in Figure 21b.

The voxel grid method for point cloud streamlining effectively reduces the number of points in the point cloud, particularly at inflection points, while ensuring a controllable down-sampling effect. This method preserves the overall characteristics of the point cloud data. Additionally, a statistical filtering process is used to eliminate noise points and outliers, preserving the structural information of the data, thus improving both the quality of the point cloud and the processing speed. The point cloud data from the cutting vertebrae path are parameterized using the cumulative chord length parameterization method. The control vertices are determined by solving a system of linear equations, with some control vertices shown in Table 7.

**Figure 22:**
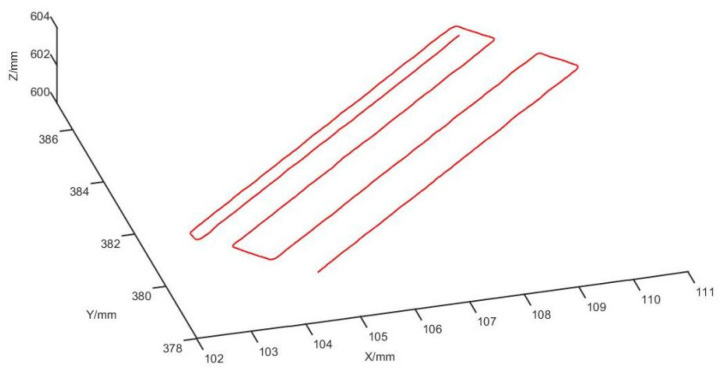
Robotic vertebral plate-cutting simulation trajectory.

After obtaining the control vertices, the cutting trajectory point cloud is fitted using the NURBS curve fitting formula, as shown in Figure 22. Once the fitting is complete, the spacing between neighboring points is determined based on the accuracy requirements for the actual cutting process of the spine plate. The NURBS curve is discretized into a point cloud to preserve the characteristics of the trajectory, which aids in the generation of the robot’s cutting trajectory.

To verify the accuracy of the trajectory planning method, the Robotics Toolbox was employed to simulate the robot’s application. The joint angles corresponding to the cutting trajectory of the vertebral plate were imported into the robot model to drive its movement. Some of these joint angles are presented in Table 8.

**Figure 23:**
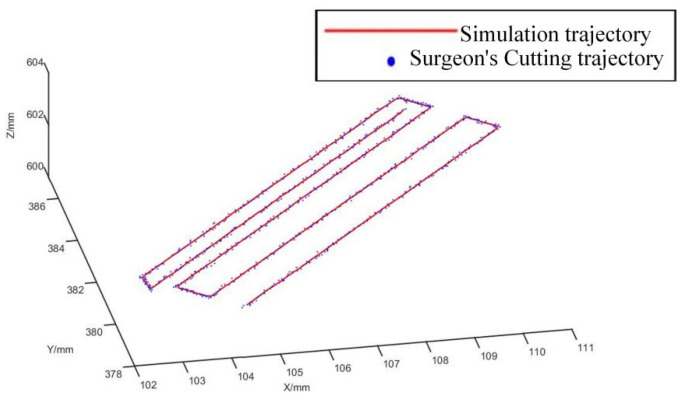
Comparison of robot and surgeon cutting trajectories.

Figure 23 presents a comparison between the simulated robot trajectory for vertebral plate cutting and the surgeon’s cutting trajectory. It demonstrates that the robot’s cutting motion is smooth with minimal fluctuation, aligning with the expected cutting trajectory and effectively replicating the trajectory of the surgeon’s procedure.

This comparison indicates that the robot can accurately follow the planned trajectory, achieving precision in vertebral plate cutting similar to that of a surgeon. The successful replication of the surgeon’s cutting trajectory by the robot underscores the effectiveness of the trajectory planning method and the potential for robotic systems to enhance the precision and efficiency of spinal surgeries.

### 3.3. Simulation Validation of Trajectory Planning Under Parameter Prediction

To assess the effectiveness of the robot trajectory planning method, which integrates trajectory parameter prediction and the cutting trajectory corner rounding processing method, a case study using a patient sample was selected. Specifically, the seventh sample listed in Table 3 was chosen for simulation experiments. Table 3 outlines the number of cutting rows and layers for the vertebral plate of this sample, which are 6 and 10, respectively. The cutting trajectory parameter information for this sample is provided in Table 9.

**Figure 24:**
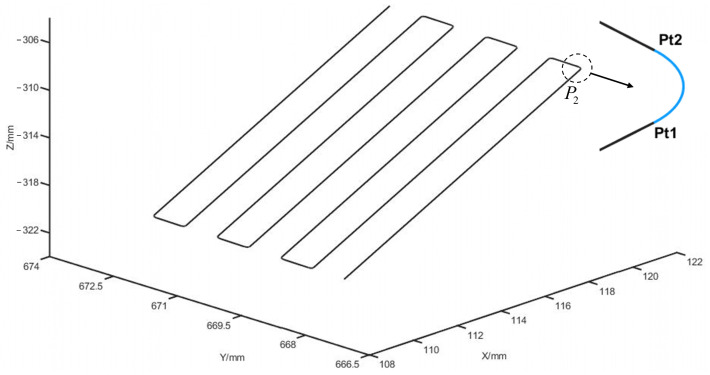
Robot cutting simulation trajectory.

Using the trajectory parameter information from Table 9, the cutting trajectory was simulated in MATLAB, as illustrated in Figure 24. This figure demonstrates that the robot trajectory planning method, based on trajectory parameter prediction, effectively generates personalized vertebrae-cutting trajectories.

In addition, the cutting trajectory corner rounding method addresses the issue of discontinuous trajectory corners. For example, at a characteristic point $P_{2}$, an arc segment between two points $Pt_{1}$ and $Pt_{2}$ is used to smooth sharp corners, thereby enhancing the trajectory’s continuity. This improves the robot’s motion efficiency by reducing frequent start–stop wear on the robot joints and other mechanical issues, thus enhancing the quality and efficiency of the vertebral plate-cutting process.

This simulation validation confirms that the proposed trajectory planning method, including corner rounding, not only ensures continuous motion but also helps optimize the robotic system’s performance during vertebral plate cutting, contributing to smoother and more efficient surgical procedures.

### 3.4. Robotic Vertebral Plate-Cutting Trajectory Planning Experiment

#### 3.4.1. Experiment Objective

The primary objective of this experiment is to validate the accuracy and adaptability of a robotic system for vertebral plate cutting, focusing on generating a precise cutting trajectory that reflects patient-specific anatomical variations. Through BP neural network-based prediction and optimized trajectory point generation, the experiment seeks to ensure that the robotic system can perform high-precision, smooth, and anatomically consistent cuts that align with the needs of individualized vertebral structures.

#### 3.4.2. Experiment Process

(1) Prediction of cutting trajectory parameters

Prior to performing robotic cutting experiments on the spine model, personalized cutting trajectory parameters need to be determined using the BP neural network prediction model. These parameters include the cutting area dimensions (length, width, and depth), the number of row spacings, and the number of layers to be cut. In order to control for randomness and ensure that the trajectory adapts to individualized anatomical variations, three sets of spine models with varying anatomical characteristics were selected for the experiments. Cutting area parameters such as length, width, and depth of these vertebral models, which can be determined by modeling software and preoperative imaging data, were used as input parameters to the BP neural network prediction model to obtain cutting trajectory output parameters such as the number of row spacing and number of cutting levels. Table 10 lists the BP neural network prediction model input and output parameters for the three sets of vertebral models, which reflect the variability inherent in individual anatomical characteristics. This is critical to ensure that the robotic system can adapt to the specific geometry of different patients, thus facilitating personalized robotic surgery.

(2) Generation of cutting trajectory points

Based on the predicted cutting trajectory parameters, a trajectory parameter-based robot trajectory planning method was utilized to generate accurate cutting trajectory points for each vertebral model. These points were optimized to ensure smooth transitions, especially around inflection points, for each trajectory to reduce stress on the cutting tool and maintain the anatomical integrity of the vertebra. Table 11 presents the trajectory points for the third group, beginning with point V001 as the designated start.

(3) Execution of cutting experiments and analysis of results

**Figure 25:**
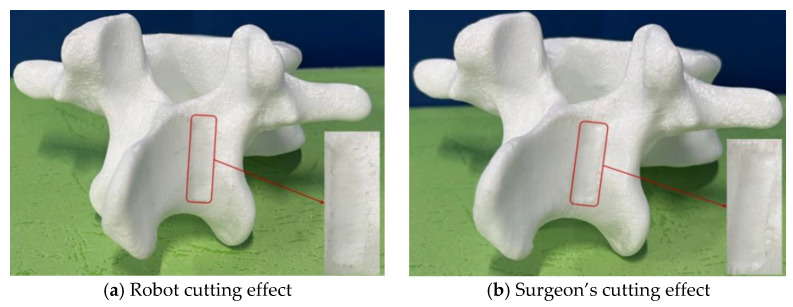
Cutting effects of the first group of vertebral body models.

**Figure 26:**
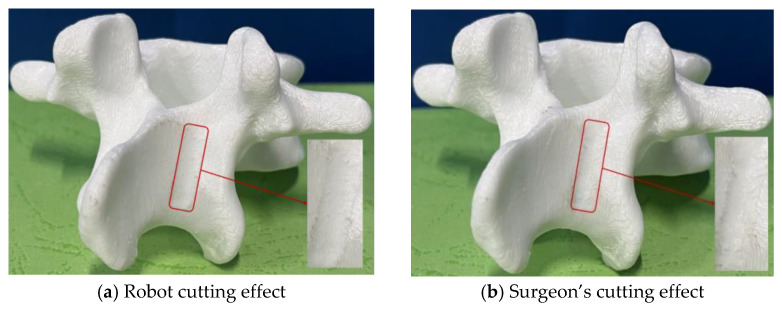
Cutting effects of the second group of vertebral body models.

**Figure 27:**
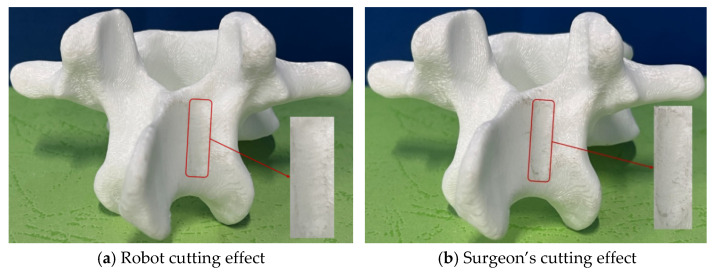
Cutting effects of the third group of vertebral body models.

Using the optimized trajectory points, the robotic cutting experiments were conducted on each vertebral model. Each cut was executed in a controlled environment, ensuring consistent tool alignment and optimized feed rates to minimize errors. Figure 25a, Figure 26a and Figure 27a illustrate the robotic cutting results for the three groups, with quantitative data recorded through high-precision optical probes and vernier calipers with an accuracy of 0.01 mm. Table 12 details the cutting area parameters as measured post-cutting, with the error values indicating deviations from the initially specified parameters.

(4) Comparative analysis with surgeon cutting results

To comprehensively assess the robotic system’s efficacy, a comparative study was conducted using three identically prepared vertebral models for manual cutting by an experienced surgeon. As shown in Figure 25b, Figure 26b and Figure 27b, the robotic cuts displayed enhanced precision with flatter surfaces, reduced edge protrusions, and a uniform cutting area, resulting in a more consistent and regular cutting profile than manual methods. This advantage is particularly evident in parameters such as smoothness, accuracy, and overall cutting efficiency, underscoring the robot’s potential for superior surgical precision.

(5) Extension to patient-specific surgery simulations

**Figure 28:**
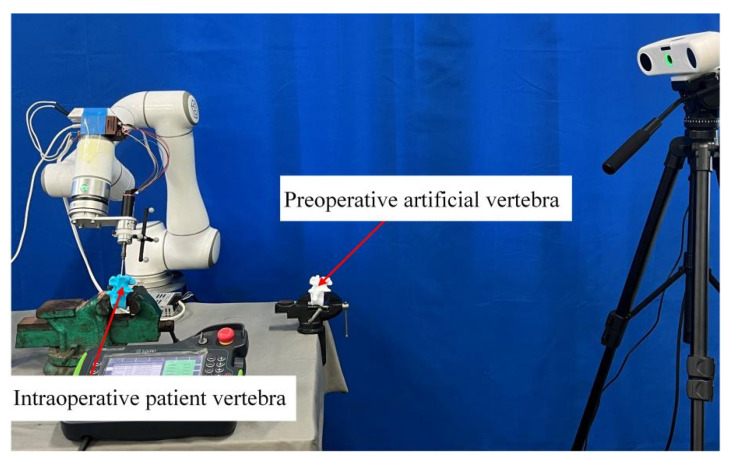
Experimental platform for robotic vertebral cutting.

**Figure 29:**
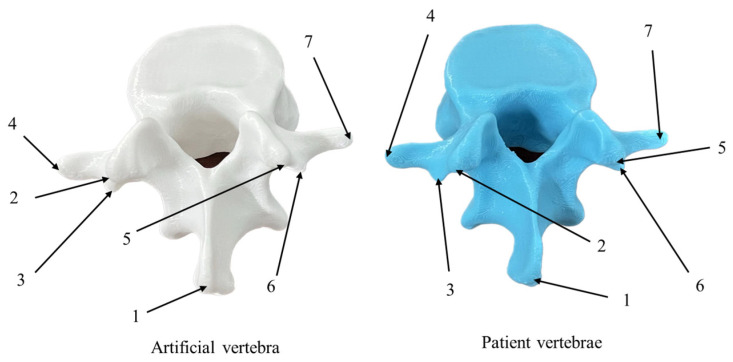
Position selection of marking points for vertebrae to be aligned.

Due to ethical constraints, 3D-printed vertebral models with patient-mimicking characteristics were employed for further experimental validation. An integrated experimental platform for robotic vertebral cutting was constructed, depicted in Figure 28, with alignment established using seven specific registration points across the artificial and patient vertebrae. These points, detailed in Table 13, were strategically selected based on anatomical landmarks such as the spinous process and mastoid process to optimize the registration process, as illustrated in Figure 29.

To ensure minimal random error, ten sets of measurements were taken for each marker point, with the averaged coordinates used to define positional relationships, as summarized in Table 14. With the registration matrix established, the robot’s cutting trajectory could be seamlessly translated from the artificial to the patient vertebrae space, allowing for precise surgical space trajectory mapping.

**Figure 30:**
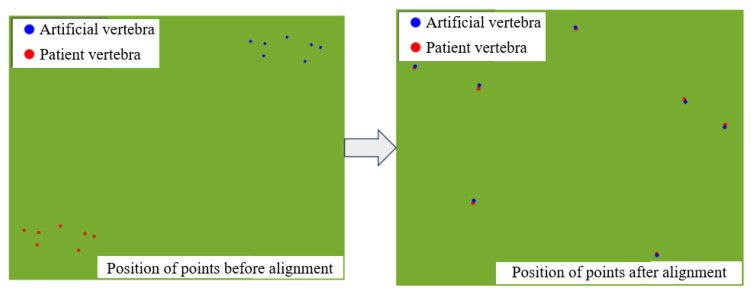
Registration results of artificial vertebrae and patient vertebrae.

**Figure 31:**
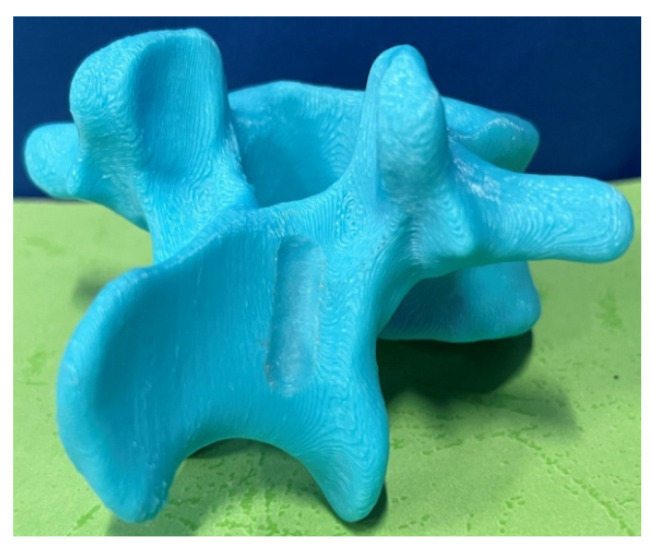
Cutting effect of patient vertebral body.

With the positional information, the registration relationship matrix Tptps between the artificial and patient vertebrae was established, and the registration results are shown in Figure 30. The cutting trajectory point cloud from the robotic space was transformed into the surgical space, and the orthopedic surgical robot’s cutting trajectory on the actual patient vertebrae was obtained. The cutting effect is illustrated in Figure 31.

(6) Cutting performance verification

Following robotic cutting, the optical probe tool and vernier calipers were again employed to validate the cutting area parameters for both the model and patient vertebrae, with the results summarized in Table 15. A close alignment between the model and patient data further confirmed the robot’s precision and reliability, with only minor deviations observed.

An analysis of Figure 31 indicates that the robotic system achieved a smooth, consistent, and accurately positioned cut on the patient vertebral plate. The absence of concave or convex distortions and continuous cutting operation without start–stop interruptions ensure enhanced safety and precision, demonstrating the robotic system’s potential to replicate surgeon-level skills through precise spatial mapping.

#### 3.4.3. Experiment Results

The experimental results reveal that the robotic vertebral plate-cutting system achieves a notable advancement in precision-guided surgical applications, with metrics reflecting enhanced spatial accuracy, consistent trajectory adherence, and optimized surface regularity. The comparative analyses with manual techniques underscore the robot’s superior performance, achieving minimal deviations in cutting dimensions and a smoother surface quality. Through patient-specific simulation using 3D-printed anatomical models, the system demonstrated reliable trajectory replication with precise anatomical alignment, supporting its potential for integration into clinical workflows. These results substantiate the system’s capability to meet the complex demands of vertebral surgery, providing a scalable solution for personalized, high-accuracy surgical interventions.

## 4. Conclusions

This paper presents the successful development and validation of a robotic vertebral plate-cutting system, designed to enhance surgical precision through advanced trajectory planning and skill-learning mechanisms. The key outcomes of the research are summarized as follows:

(1) Accurate Spatial Mapping and Alignment: The spatial mapping relationship between the surgeon’s cutting trajectory and the robot’s movement was successfully established. This relationship was verified through rigorous calibration, ensuring the required precision for surgical localization. The system demonstrated the capability to accurately replicate the surgeon’s cutting technique, providing a robust foundation for high-precision robotic-assisted surgery.
(2) Personalized Trajectory Planning: By using point cloud data from robot cutting simulations on a 3D-printed vertebral model, personalized cutting trajectories were successfully planned. The data underwent preprocessing, NURBS curve fitting, and parametric discretization, resulting in the accurate mapping of the surgeon’s cutting path into the robotic coordinate space. The simulation results showed that the robot could effectively reproduce the planned trajectory with high fidelity.
(3) Trajectory Parameter Prediction with BP Neural Network: A BP neural network model was developed to predict the cutting trajectory parameters based on historical data. The model’s high accuracy and stability were validated through training and simulation, allowing for the generation of personalized cutting trajectories tailored to individual patient anatomy.
(4) Experimental Validation and Improvement in Cutting Precision: The experimental trials using artificial vertebral models and patient vertebrae confirmed the effectiveness of the robotic system. The registration methods such as PCA + ICP ensured alignment between the artificial and real vertebrae, while the robot-assisted cutting experiments showed significant improvements in cutting quality and precision compared to traditional methods.

This research underscores the potential of skill-learning-based personalized trajectory planning in robotic surgery. The results demonstrate a marked improvement in the precision and stability of robotic vertebral plate cutting, advancing the capabilities of orthopedic robotic surgery and laying the groundwork for safer, more effective surgical interventions.

Although this study successfully developed and validated a robotic vertebral plate-cutting system, improving cutting accuracy and stability, there are still some limitations. First, the artificial vertebral models differ from biological tissues, which may impact system precision, especially with interference from blood, soft tissues, and other factors. Second, the optical positioning system’s robustness against interference and the protection of critical structures still require further research to ensure precision in complex environments. Lastly, despite the promising experimental results, further validation in biological environments or preclinical settings is necessary to assess the system’s stability and safety. Future work will focus on enhancing the system’s dynamic adaptability, optimizing sensor technologies, and conducting biological model validation to support clinical application.

## Figures and Tables

**Table 1:** Combination of transfer functions.

Groups	Implicit Layer	Output Layer
1	logsig	logsig
2	logsig	tansig
3	logsig	purelin
4	tansig	logsig
5	tansig	tansig
6	tansig	purelin

**Table 2:** Parameters of input and output quantities for the BP neural network model.

Samples	Individual Characteristic			Input Quantity		Output Quantity	
	Age	Gender	Disease Course (Months)	Cutting Width	Cutting Depth	Numbers of Row	Numbers of Layer
				$X_{0}$ (mm)	$D_{0}$ (mm)	h	*c*
1	42	Male	13	4.6	3.5	6	9
2	49	Male	18	5.1	3.9	5	10
3	51	Female	14	4.7	3.7	6	9
4	55	Male	23	5.5	4.0	7	10
5	56	Female	20	5.2	3.9	7	10
6	67	Male	27	6.0	4.5	9	11

**Table 3:** Prediction results of the BP neural network model.

Samples	Regional Scope		Experimental Values		Predicted Values	
	Width $X_{0}$ (mm)	Depth $D_{0}$ (mm)	Number of Rows h	Number of Layers c	Number of Rows $h_{1}$	Number of Layers $c_{1}$
1	6.3	4.2	8	11	8	11
2	5.4	4.2	6	11	6	11
3	6.1	3.8	8	10	8	10
4	5.4	4.3	7	11	7	11
5	4.8	3.6	6	9	6	9
6	6.2	3.6	9	9	9	9
7	4.8	4.2	6	10	6	10
8	5.5	3.5	7	9	7	9
9	6.3	4.4	9	11	9	11
10	5.1	4.3	8	11	8	11
11	6.3	4.0	7	11	7	11
12	5.5	3.6	7	9	6	9
13	5.1	3.9	5	10	5	10

**Table 4:** Calibration point coordinates in the optical measurement coordinate system.

Groups	*X* (mm)	*Y* (mm)	*Z* (mm)	*Rx* (°)	*Ry* (°)	*Rz* (°)
1	104.137	−175.988	1455.654	−3.385	17.960	−96.386
2	168.952	−166.611	1435.620	−14.979	27.659	−92.772
3	199.542	−196.824	1461.806	2.580	15.479	−86.001
4	137.640	−155.995	1512.582	4.484	15.157	−91.378
5	109.137	−149.509	1603.012	−2.108	16.167	−96.343
6	217.360	−171.054	1647.207	6.044	10.588	−102.169

**Table 5:** Calibration point coordinates in the robot base coordinate system.

Groups	*X* (mm)	*Y* (mm)	*Z* (mm)	*Rx* (°)	*Ry* (°)	*Rz* (°)
1	348.317	559.148	42.466	6.753	−15.144	2.158
2	365.937	637.438	52.916	−7.146	−6.083	7.041
3	458.643	783.039	208.856	6.789	−18.862	12.146
4	357.311	721.249	178.649	11.876	−18.637	6.671
5	381.147	590.236	197.317	8.094	−16.919	1.874
6	517.903	479.448	203.707	19.919	−21.929	−5.220

**Table 6:** Results of accuracy verification of spatial mapping relationship.

Groups	Target Position (*x*, *y*, *z*) (mm)	Actual Position (*x*′, *y*′, *z*′) (mm)	Deviation (mm)
1	(−103.309, 21.796, 1144.483)	(−103.118, 22.214, 1144.169)	0.557
2	(−153.929, 32.368, 1150.065)	(−153.577, 32.121, 1150.253)	0.469
3	(−107.551, 102.544, 1142.223)	(−107.863, 102.198, 1142.401)	0.498
4	(−120.067, 82.053, 1171.539)	(−120.411, 81.696, 1171.244)	0.577
5	(−137.513, 134.355, 1214.150)	(−137.057, 133.986, 1214.373)	0.628
6	(−95.237, 113.483, 1342.117)	(−94.793, 113.835, 1341.899)	0.607
7	(−135.509, 131.949, 1271.236)	(−135.818, 132.232, 1270.858)	0.564
8	(−155.591, 108.775, 1315.822)	(−155.122, 109.134, 1315.597)	0.632
9	(−88.279, 159.660, 1269.717)	(−88.617, 159.336, 1269.878)	0.495
10	(−72.944, 130.329, 1175.044)	(−73.213, 129.910, 1175.288)	0.554

**Table 7:** Six sets of control vertices for the point cloud.

Point Cloud Number	Control Vertices (mm)
1	[105.326,379.014,601.111]
2	[105.474,379.157,601.212]
3	[105.705,379.402,601.319]
4	[105.841,379.613,601.393]
5	[105.953,379.833,601.472]
6	[106.149,380.010,601.578]

**Table 8:** Robot joint angles (unit: radians).

Joint 1	Joint 2	Joint 3	Joint 4	Joint 5	Joint 6
1.332	−1.105	1.578	3.875	1.212	2.197
1.342	−1.094	1.560	3.885	1.274	2.207
1.344	−1.084	1.544	3.891	1.268	2.208
1.333	−1.096	1.564	3.880	1.272	2.198
1.322	−1.108	1.583	3.870	1.275	2.188
1.334	−1.087	1.550	43.886	1.272	2.199

**Table 9:** Partial list of cutting trajectory parameters of sample vertebral plates.

Characteristic Points	Coordinate Values (mm)
$P_{1}$	(110.618, 668.007, −320.904)
$P_{2}$	(118.309, 667.952, −310.411)
$k_{11}$	(118.311, 668.678, −310.073)
$k_{12}$	(110.622, 668.732, −320.566)
$k_{13}$	(110.623, 669.457, −320.228)
⋯	⋯
$P_{4}$	(110.634, 672.357, −318.875)
$P_{3}$	(118.324, 672.302, −308.382)

**Table 10:** Three sets of BP neural network prediction model input and output parameters.

Groups	Input Parameters			Output Parameters	
	Cutting Length (mm)	Cutting Width (mm)	Cutting Depth (mm)	Number of Row Spacing	Number of Cutting Layers
1	19.21	6.00	4.30	9	11
2	18.80	5.30	4.50	7	12
3	19.07	5.10	3.90	6	10

**Table 11:** Partial list of cutting trajectory points for third vertebra model.

Cutting Trajectory Point V	Coordinate Value (mm)
V001	(208.628, 696.817, −331.832)
V002	(207.989, 705.871, −346.980)
V003	(207.836, 705.876, −346.985)
V004	(207.831, 705.950, −347.109)
V005	(207.330, 705.969, −347.131)
V006	(207.337, 705.894, −346.997)
V007	(207.192, 705.903, −347.004)

**Table 12:** Robotic cutting area parameters and associated errors.

Groups	Cutting Length (mm)	Cutting Width (mm)	Cutting Depth (mm)	Errors (mm)
1	19.52	5.63	4.06	0.31/0.37/0.24
2	18.55	4.97	4.27	0.25/0.33/0.23
3	18.80	5.46	3.71	0.27/0.36/0.19

**Table 13:** Anatomical landmarks used for vertebral registration.

Points	1	2,5	3,6	4,7
Position	Spinous process	Mastoid process	Protrusion	Transverse process

**Table 14:** Measurements of vertebral registration points.

Marker Point Number	Model Marking Points $p_{s}$ $\left(x_{s},\;y_{s},\;z_{s}\right)$ (mm)	Patient Marking Points $p_{t}$ $\left(x_{t},\;y_{t},\;z_{t}\right)$ (mm)
1	(230.904, 145.653, 1220.740)	(−3.670, −31.413, 1146.899)
2	(206.896, 128.167, 1246.129)	(−27.682, 48.901, 1172.287)
3	(208.179, 139.611, 1252.486)	(−26.398, 37.457, 1178.644)
4	(193.239, 141.726, 1256.713)	(−41.338, 35.343, 1182.872)
5	(249.753, 122.943, 1243.291)	(15.177, −54.124, 1169.450)
6	(256.179, 138.537, 1247.235)	(21.602, −38.532, 1173.393)
7	(265.714, 135.979, 1253.130)	(31.139, −41.088, 1179.289)

**Table 15:** Measured parameters of cutting area on model and patient vertebrae.

Parameters	Model Cutting Area	Patient Cutting Area	Errors (mm)
Length (mm)	18.96	18.29	0.67
Width (mm)	5.94	5.53	0.41
Depth (mm)	4.22	4.49	0.27

## Data Availability

All the data generated or analyzed during this study are included in this published article.
